# Serum Metabolic Fingerprints Characterize Systemic Lupus Erythematosus

**DOI:** 10.1002/advs.202304610

**Published:** 2023-11-12

**Authors:** Shunxiang Li, Huihua Ding, Ziheng Qi, Jing Yang, Jingyi Huang, Lin Huang, Mengji Zhang, Yuanjia Tang, Nan Shen, Kun Qian, Qiang Guo, Jingjing Wan

**Affiliations:** ^1^ School of Biomedical Engineering and Med‐X Research Institute Shanghai Jiao Tong University Shanghai 200030 P. R. China; ^2^ State Key Laboratory for Oncogenes and Related Genes Shanghai Key Laboratory of Gynecologic Oncology and Department of Obstetrics and Gynecology Renji Hospital School of Medicine Shanghai Jiao Tong University Shanghai 200127 P. R. China; ^3^ Department of Rheumatology and Shanghai Institute of Rheumatology Renji Hospital School of Medicine Shanghai Jiao Tong University Shanghai 200001 P. R. China; ^4^ School of Chemistry and Molecular Engineering East China Normal University Shanghai 200241 P. R. China; ^5^ Shanghai Institute of Thoracic Tumors Shanghai Chest Hospital Shanghai Jiao Tong University Shanghai 200030 P. R. China

**Keywords:** diagnostics, mass spectrometry, metabolites, systemic lupus erythematosus

## Abstract

Metabolic fingerprints in serum characterize diverse diseases for diagnostics and biomarker discovery. The identification of systemic lupus erythematosus (SLE) by serum metabolic fingerprints (SMFs) will facilitate precision medicine in SLE in an early and designed manner. Here, a discovery cohort of 731 individuals including 357 SLE patients and 374 healthy controls (HCs), and a validation cohort of 184 individuals (SLE/HC, 91/93) are constructed. Each SMF is directly recorded by nano‐assisted laser desorption/ionization mass spectrometry (LDI MS) within 1 minute using 1 µL of native serum, which contains 908 mass to charge features. Sparse learning of SMFs achieves the SLE identification with sensitivity/specificity and area‐under‐the‐curve (AUC) up to 86.0%/92.0% and 0.950 for the discovery cohort. For the independent validation cohort, it exhibits no performance loss by affording the sensitivity/specificity and AUC of 89.0%/100.0% and 0.992. Notably, a metabolic biomarker panel is screened out from the SMFs, demonstrating the unique metabolic pattern of SLE patients different from both HCs and rheumatoid arthritis patients. In conclusion, SMFs characterize SLE by revealing its unique metabolic pattern. Different regulation of small molecule metabolites contributes to the precise diagnosis of autoimmune disease and further exploration of the pathogenic mechanisms.

## Graphical abstract

Systemic lupus erythematosus (SLE) with the highest area under the curve value of 0.992 by sparse learning of serum metabolic fingerprints of a large cohort of 915 individuals are characterized. A high‐performance nano‐assisted laser desorption/ionization mass spectrometry is developed for the acquisition of serum metabolic fingerprints within 1 min using 1 µL of each native serum. This work provides a promising assay for SLE precision diagnosis with high throughput and low sample consumption in clinics.

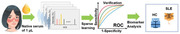

## 1 Introduction

Systemic lupus erythematosus (SLE) is a heterogeneous autoimmune disease with diverse clinical manifestations and unpredictable disease courses, which affects more than 6 million people in the world.^[^ ^1^ ^]^ The various organ involvements of SLE patients cause a profound effect on their health‐related life quality, the delayed diagnosis of which would lead to organ damage accrual and retard the survival improvement.^[^ ^2^ ^]^ However, due to the complexity and heterogenicity of SLE,^[^ ^3^ ^]^ the underlying pathogenic mechanism has not been fully elucidated, restricting the discovery of reliable biomarkers for its diagnosis and related metabolic pathway analysis.

Metabolic fingerprints correlate with other omics (e.g., genomics and proteomics), as metabolites are the end products of gene expression.^[^ ^4^ ^]^ Addressing the delayed diagnosis and high cost of current genomic and proteomic biomarkers,^[^ ^5^ ^]^ metabolic biomarkers provide a more distal characterization of pathological and physiological processes, which are more sensitive to the slight variations of health status.^[^ ^6^ ^]^ In addition, the translational use of metabolic biomarkers in concert with genomic and proteomic markers may change the way of biomarker utility in SLE. However, the manner how SLE hallmarks (metabolic disorders, organ injuries, and autoimmunity abnormality, etc.) affect metabolites is still unknown.^[^ ^7^ ^]^ The limited exploration of SLE metabolic fingerprint attributes to two key factors: 1) lack of a large sample cohort to exclude the individual difference, and 2) absence of advanced metabolic detection tool.

**Scheme 1:**
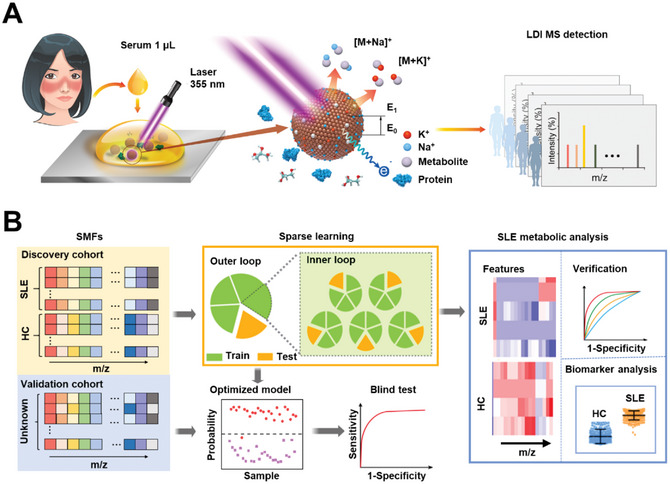
Nano‐assisted acquisition of serum metabolic fingerprints (SMFs) and systemic lupus erythematosus (SLE) diagnosis by sparse learning. A) Experimental process of laser desorption/ionization mass spectrometry (LDI MS) detection. 1 µL of native serum per individual was mixed with ferric particles for LDI MS detection. The mass to charge (*m/z)* features of sodium ion (Na^+^) and potassium ion (K^+^) adducts were recorded under the irradiation of Nd:YAG laser (355 nm). B) Sparse learning of SMFs was conducted for SLE metabolic analysis. The SMFs of SLE patients and healthy controls (HCs) in the discovery cohort were first applied in 5‐fold cross‐validation with 20 rounds, yielding 100 diagnostic models for verification of receiver operating characteristic (ROC) curves. The optimized model was obtained by assessing ROC curves of above 100 models. Then an independent validation cohort was applied to the optimized model to obtain the blind test result. Specific *m/z* features were also screened out as biomarkers and constructed as a biomarker panel for analysis.

Serum detection assays promise SLE diagnostics owing to their minimal invasiveness and desirable adaptability for large‐scale clinic use, superior to the conventional methods (e.g., biopsy and physical examination).^[^ ^8^ ^]^ Current analytic tools for serum metabolic analysis mainly include nuclear molecular resonance (NMR) spectroscopy and mass spectrometry (MS).^[^ ^9^ ^]^ Superior to the NMR of suboptimal sensitivity and limited identification capability, MS affords high sensitivity and favorable biomarker identification ability assisted by tandem MS. Mainly, laser desorption/ionization (LDI) MS enables fast analysis speed, low sample consumption, and cost‐effective expenses by nano‐assisted solid‐gas transition,^[^ ^4^, ^10^ ^]^ promising to be a powerful analytical tool in the coming era of precision medicine. Herein, we acquired serum metabolic fingerprints (SMFs) of 915 individuals by nano‐assisted LDI MS (**Scheme** **1**A), which could be deciphered by sparse learning for SLE diagnosis and metabolic biomarker panel construction (Scheme 1B).

## 2 Experimental Section

### 2.1 Study Design and Population

**Figure 1:**
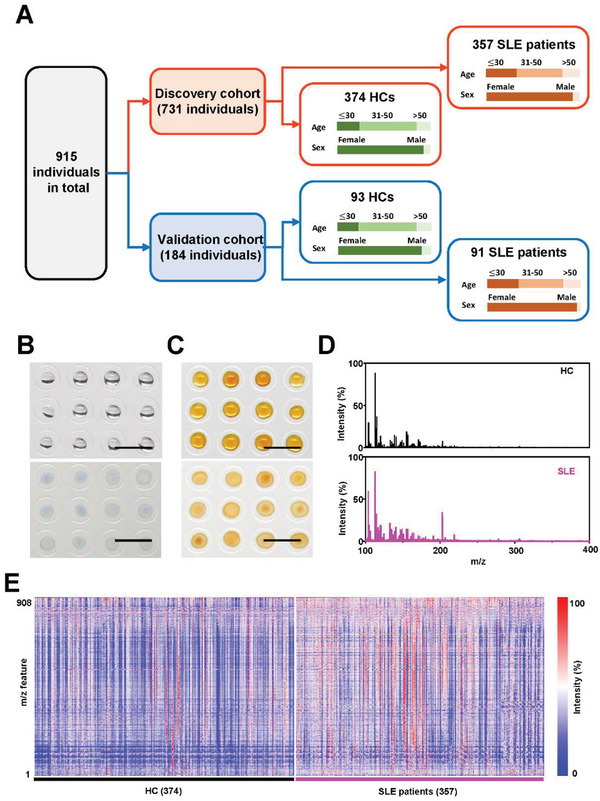
SMF extraction by nano‐assisted LDI MS. A) Sample characteristics and study design of 915 individuals, including 731 in the discovery cohort and 184 in the validation cohort. The SMF acquisition process is shown in (B,C). B) The serum samples were collected from SLE patients and HCs according to standard protocols (see Methods for more details) with only 1 µL of serum per individual loaded on the plate. C) The matrix suspension was mixed with serum sample for direct LDI MS detection. D) Typical MS spectra of an SLE patient and a healthy volunteer with *m/z* of 100–400. E) The blueprint consists of 731 SMFs in the discovery cohort, each of which contains 908 *m/z* features.

This is a cross‐sectional study with a total of 915 individuals, including 448 SLE patients and 467 healthy controls (HCs) (**Figure** **1**A and Tables S1 and S2, Supporting Information). All the patients were recruited from Renji Hospital, School of Medicine, Shanghai Jiao Tong University, from Oct. 1^st^, 2016 to Jun. 30^th^, 2018. All SLE patients fulfilled the classification criteria of 2012 systemic lupus international collaborating clinics (SLICC).^[^ ^11^ ^]^ For the HCs, 467 healthy volunteers, who showed no signs of arthralgia, heart failure, renal failure, autoimmune disease, inflammatory conditions, and other major diseases, were included in this study. All the participants gave their written informed consents before the beginning of the study. This research was conducted in accordance with the Declaration of Helsinki and approved by the institutional ethics committee of Renji Hospital (RA‐2019‐156), School of Medicine, Shanghai Jiao Tong University.

Specifically, 731 individuals (357 SLE patients and 374 age‐ and sex‐matched HCs) were randomly employed as the discovery cohort for SLE diagnostic model building (Table S1, Supporting Information). The other 184 individuals (91 SLE patients and 93 age‐ and sex‐matched HCs) were applied as the independent validation cohort for verifying the SLE diagnostic model (Table S1, Supporting Information). No significant differences of age and sex were discovered between the discovery and validation cohort for SLE patients, ensuring the effective validation results (*p* > 0.05, Table S2, Supporting Information).

The organ involvements of 448 SLE patients were confirmed based on their medical history and pathological examinations, including 228 with renal involvement, 203 with mucocutaneous involvement, 134 with hematological involvement, 123 with musculoskeletal involvement, and 87 with cardiorespiratory involvement. In addition, a small RA queue was collected for disease controls, and a separate queue (SLE/HC, 14, 13) for metabolic marker detection under the same ethics committee (RA‐2019‐156). All the participants gave their written informed consents before the beginning of the study.

This research was conducted following the Declaration of Helsinki and approved by the institutional ethics committee of Renji Hospital (RA‐2019‐156), School of medicine, Shanghai Jiao Tong University.

## 3 Results

### 3.1 Serum Metabolic Fingerprint Analysis

We carried out the metabolic measurement of 1 µL of native serum from each sample in a microarray manner (Figure 1B), without any complex pretreatment. For a typical LDI MS detection, we loaded the serum sample microarray with ferric particles as the matrix (see Methods in Supporting Information, Figure 1C). Notably, the ferric particles demonstrated nanoscale surface roughness for selective metabolite enrichment and stable crystalline structure eliminating the conventional sweet‐spot searching (Figure S1, Supporting Information). These ferric particles can be ideal for LDI MS due to the fine water dispersity (polydispersity index (PDI) < 0.3, Figure S2A, Supporting Information) for matrix use, negative surface charge (zeta potential, Figure S2B, Supporting Information) for cation adduct,^[^ ^4^, ^8^, ^12^ ^]^ strong light absorption in the ultraviolet range (Figure S2C, Supporting Information) for laser energy transfer during LDI process.^[^ ^13^ ^]^ Besides, the preparation of ferric particles is scalable and capable of > 150 000 tests per batch (Figure S2D, Supporting Information) since only 1 µg of ferric particles is required per LDI MS detection (Figure 1C). Consequently, nano‐assisted LDI MS achieved the original data acquisition within 1 minute per individual. The typical MS spectra acquired for SLE and HC were exhibited in Figure 1D.

A blueprint of SLE patients and HCs displayed in Figure 1E summarized the SMFs of 731 individuals (SLE/HC, 357/374) in the discovery cohort. Notably, the mass to charge (*m/z*) features were focused on the low mass range of small metabolites (*m/z* of 100–1000), considering the desirable detection selectivity (dealing with high concentrations of salts/proteins in Figure S3, Supporting Information) and sensitivity (dealing with low concentrations of small metabolites in Figure S4, Supporting Information). Specifically, from the raw mass spectrum containing ≈ 120 000 *m/z* data points per sample, the SMF of 908 *m/z* features was obtained by searching the local maxima. We also examined the intra‐similarity of mass spectra using cosine correlation analysis, confirming the high similarity within the SLE/HC group (≈90% SLE patients/HCs with similarity score > 0.9, Figure S5, Supporting Information). Therefore, our platform features fast analytical speed (< 1 minute per individual) and high‐throughput (908 *m/z* features within the ≈120 000 data points of raw mass spectra) for achieving the large cohort extraction of SMFs, laying the solid foundation for following the SLE diagnostic model construction.

### 3.2 Diagnosis by Machine Learning

**Figure 2:**
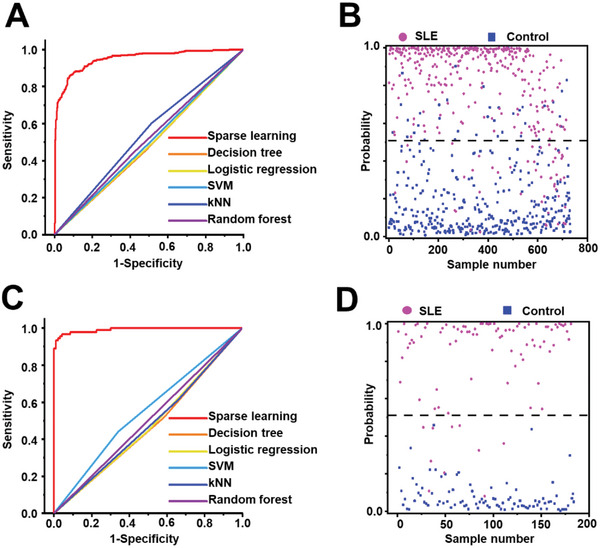
Differentiation of SLE from HCs by machine learning methods. A) ROC curves based on the 731 SMFs (SLE/HC, 357/374) in the discovery cohort, using sparse learning (AUC of 0.950), decision tree (AUC of 0.486), logistic regression (AUC of 0.489), supporting vector machine (SVM, AUC of 0.498), K‐nearest neighbors (kNN, AUC of 0.544), and random forest (AUC of 0.513). B) The scatter plot of probability in the discovery cohort is based on the optimized diagnostic model of sparse learning. C) ROC curve based on the 184 SMFs (SLE/HC, 91/93) in the validation cohort, using sparse learning (AUC of 0.992), decision tree (AUC of 0.533), logistic regression (AUC of 0.527), SVM (AUC of 0.450), kNN (AUC of 0.523), and random forest (AUC of 0.499). D) The scatter plot of probability in the validation cohort is based on the optimized diagnostic model of sparse learning. Every dot in (B) and (D) represents one individual in this study.

We diagnosed SLE patients from HCs by machine learning of the SMFs (**Figure** **2**). Based on the sufficient sample size demonstrated by power analysis (Figure S6, Supporting Information),^[^ ^14^ ^]^ we studied the diagnostic performance of SMFs in identifying SLE patients from HCs by using different machine learning methods. In the discovery cohort, the sparse learning of SMFs achieved the diagnostic AUC of 0.950 with a 95% confidence interval (CI) of 0.935‐0.965 (Figure 2A and Table S3, Supporting Information), the performance of which remained stable as changing model numbers (Figure S7 and Table S4, Supporting Information). The employed parameters of the sparse learning were determined by the iterative optimization process (Figure S8, Supporting Information), which was based on the literature with slight modifications.^[^ ^8a^ ^]^ Notably, the SLE patients could be differentiated from HCs with a sensitivity/specificity of 86.0%/92.0% (Figure 2B). In contrast, other machine learning methods only afforded the limited AUC of 0.486‐0.544 (*p* < 0.05, Figure 2A, and Table S3, Supporting Information).

We also conducted the machine learning methods on the SMFs of an independent validation cohort. Specifically, sparse learning of SMFs achieved the diagnostic AUC of 0.992 with 95% CI of 0.983‐1.000 for diagnosing SLE patients from HCs, higher than the AUC of 0.450‐0.533 afforded by other machine learning methods (*p* < 0.05, Figure 2C, and Table S3, Supporting Information). Accordingly, SMFs by sparse learning achieved the sensitivity/specificity of 89.0%/100.0% (Figure 2D), much higher than other machine learning methods (sensitivity/specificity of 44.1%−61.3%/34.1%−65.9%, *p* < 0.05, Table S3, Supporting Information). We also investigated the performance of major organ involvements in SLE (Table S5, Supporting Information), and the results showed limited specificity. This could be due to the fact that the majority of SLE patients have multi‐organ involvement (Figure S9, Supporting Information), and the biomarkers selected based on multi‐organ involvement have limited effectiveness in distinguishing patients with involvement of different organs. For disease activity, while the SMFs failed to distinguish the SLE patients with low disease activity (SLEDAI ≤ 6) and high disease activity (SLEDAI > 6), the established model maintained the diagnostic performance (AUC of 0.898) in identifying the SLE patients with low disease activity (Figure S10, Supporting Information). Briefly, the superiority of SMFs by sparse learning has been demonstrated for SLE diagnosis due to its high consistency exhibited in the discovery and validation cohort.

### 3.3 Construction of SLE Metabolic Biomarker Panel

**Figure 3:**
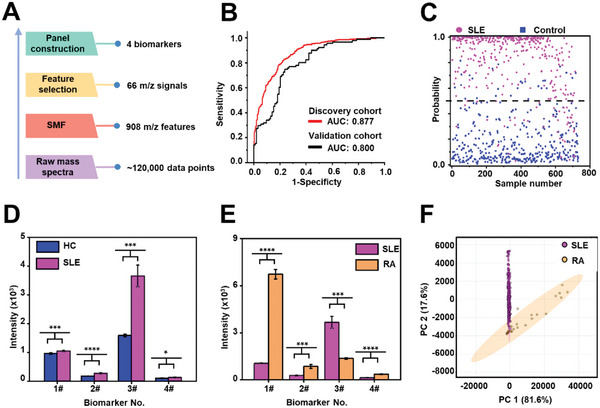
Construction of metabolic biomarker panel of SLE. A) Workflow for constructing the metabolic biomarker panel. After the pretreatment of raw mass spectra (containing 120 000 data points), the SMF of 908 *m/z* features was extracted, yielding 66 *m/z* signals through the feature selection process, thus identifying 4 biomarkers. B) The ROC curves by sparse learning of the 4 biomarkers in the discovery cohort (red line) and validation cohort (black line). C) The scatter plot of probability in discovery cohort based on the optimized diagnostic model of SMFs. D) The intensity plot of SLE patients (in pink) and HCs (in green) is based on the 4 biomarkers, according to five independent LDI MS experiments. E) The intensity plot of 357 SLE patients (in pink) and 27 rheumatoid arthritis (RA) patients (in yellow) based the 4 biomarkers, according to five independent LDI MS experiments (* represents *p* < 0.05, ** represents *p* < 0.01, *** represents *p* < 0.001, and **** represented *p* < 0.0001). F) The scores plot of SLE patients and RA patients by principal component analysis (PCA).

We expected to identify the unique metabolic pattern of SLE patients from the massive features, thus providing insights for elucidating related pathological mechanisms. We identified a biomarker panel of 4 metabolites (imidazoleacetic acid, 2‐hydroxyadipic acid, glucose, and pseudouridine) for SLE based on the optimized diagnostic model by measuring the contribution of each *m/z* feature within SMFs (**Figure** **3**A and Table S6, Supporting Information).

The identification and validation for the 4 high‐contribution *m/z* features within SMFs were conducted by an LC‐ESI‐HRMS^2^ metabolic analysis for an independent validation cohort (SLE/HC, 14/13). Specifically, the information on *m/z* and fold changes of intensities can serve as identifier to match the *m/z* features within different mass spectrometry platform of the same compound. Next, the high‐contribution *m/z* features within LDI‐MS were annotated to metabolites (imidazoleacetic acid, 2‐hydroxyadipic acid, glucose and pseudouridine) according to the matched *m/z* features within LC‐MS/MS via accurate mass and MS/MS matching with the human metabolome database (https://hmdb.ca, Table S7, Supporting Information).^[^ ^15^ ^]^ Meanwhile, the LC‐MS/MS result, consistent with the LDI‐MS result, verified that the biomarkers were reliable.

Sparse learning of biomarker panel reached an enhanced diagnostic AUC of 0.800–0.877 (Figure 3B,C, and Table S8, Supporting Information). It was critical to combine the 4 metabolic biomarkers as a biomarker panel for presenting the unique metabolic pattern of SLE, as these biomarkers showed limited diagnostic performances when singly applied (AUC of 0.66‐0.81/0.58‐0.83 in discovery/validation cohort, Figure S11 and Table S9, Supporting Information). Notably, the 4 potential biomarkers did not show correlation with SLEDAI, which suggested that the biomarkers are geared towards the diagnosis of SLE patients but have limited capability in assessing disease activity (Table S10, Supporting Information). Moreover, the validity of the above metabolic panel (4 metabolic biomarkers) was illustrated by maintaining the diagnostic performance of whole profiling of SMFs (908 *m/z* features) with slight AUC loss (<0.1, Figure 2).

In patients with rheumatoid arthritis (RA), features associated with systemic lupus erythematosus (SLE) are commonly observed and both RA and SLE are systemic autoimmune diseases, sharing some similar clinical features and underlying pathogenesis.^[^ ^16^ ^]^ We also investigated the potential metabolic biomarkers regarding the variations in SLE patients compared to HCs or a small cohort of rheumatoid arthritis (RA) patients as disease controls. Compared to HCs, these four potential biomarkers were all up‐regulated in SLE patients by affording the fold change of 1.09‐2.30 (Figure 3D, Figure S12 and Tables S11 and S12, Supporting Information). The established metabolic biomarkers showed significant differences between SLE and RA patients (357/27, Figure 3E, Tables S13 and S14, Supporting Information, *p* < 0.05). Also, the SLE and RA patients could be assembled into two clusters by applying the unsupervised cluster method of principal component analysis (PCA) (Figure 3F, Tables S13 and S14, Supporting Information), suggesting their different metabolic patterns in distal metabolic fingerprints. As the medication of RA and SLE was significantly different, we applied the propensity score matching (PSM) to select SLE patients to match the RA patients, which was conducted at a ratio of 1:1 for age, gender, and medication (prednisone, methotrexate, leflunomide, and hydroxychloroquine, Table S15, Supporting Information). The PCA analysis demonstrated the limited influence of medication. A similar result was also obtained on 20 patients (SLE/RA, 10/10) with naïve treatment (Figure S13, Supporting Information). Considering the small sample size of RA patients, the effect of medication regimens (e.g., corticosteroids) still calls for more future efforts on cohort construction with strict enrollment criteria.

We constructed a small cohort to evaluate the effect of medications on the diagnostic performance of SMFs in our SLE patients (Table S16, Supporting Information). The diagnostic model based on the SLE patients who were treatment naive and HCs showed the AUC of 0.947 (Figure S14A, Supporting Information), which maintained the major diagnostic performance based on the large cohort of 915 individuals (AUC of 0.950). The difference in medication exposure could not result in the differentiation between SLE patients (AUC of 0.504, Figure S14B, Supporting Information), indicating the limited influence of medication usage in the SLE diagnostic model. We conducted PCA analysis on SLE patients who received different dosages of corticosteroids in the discovery cohort, including 15 individuals who received no corticosteroids, 100 individuals with a daily dosage of less than 20 mg, 104 individuals with a daily dosage between 20 and 40 mg, and 79 individuals with a daily dosage greater than 40 mg. As shown in Figure S14C (Supporting Information), no distinct clusters were formed for patients with different medication scenarios, illustrating the minor role of corticosteroids in the SLE diagnostic model. Moreover, there were 27 SLE patients who exhibited complications with diabetes and dyslipidemia, the effect of which on the diagnostic model could be negligible, as shown in Figure S14D (Supporting Information). While the above analysis provided indirect insights into medication and complication effects, it is critical to construct a cohort with strict enrollment criteria to rule out the medication effect in the future.

## 4 Discussion

SLE is a progressive autoimmune disease with great heterogeneity. The accurate and in‐time diagnosis of SLE is indispensable for effective treatment and appropriate prognosis. Currently, the clinical diagnosis of SLE relies on three major classification criteria (EULAR/ACR‐2019, SLICC‐2012, and ACR‐1997 criteria) with both clinical criteria and immunological criteria involved. Besides the complexity, there were about 25.6‐30.5% of patients missed as estimated despite the major classification criteria afforded the high diagnostic performance (sensitivity of 85.7‐91.3% and specificity of 93.0‐97.3%).^[^ ^17^ ^]^ Meanwhile, it is of intense research efforts to develop the alternatives to current classification criteria based on a simple blood test, mainly focusing on the design of assays from nucleic acids and proteins.^[^ ^5^, ^18^ ^]^ Importantly, given a designed cohort like genomic/proteomic approaches to study large series of individuals (> 1000), metabolic approaches would be the next‐generation diagnostic tools, considering that 1) metabolites at the end of pathways reveal the real‐time status of patients with precision; 2) metabolic assay construction is facile and free of expensive or tedious sequencing/immunoassays.

Notably, the metabolic analysis of metabolites as end‐products has exhibited great potential for profiling complex diseases like cancers. In this study, we conducted the serum metabolic analysis for SLE patients and achieved the identification of SLE patients from HCs with a diagnostic sensitivity of 86.0–89.0% and specificity of 92.0‐100.0% in the cohort of 915 individuals (SLE/HC of 448/467). Compared with the prior serum metabolomic studies in SLE (Table S17, Supporting Information), the present study afforded the optimized diagnostic performance and improved credibility due to the advantages in sample volume and study design, detection platform, and statistical algorithms.

The sample volume and study design are of fundamental significance to the metabolic analysis. In this study, a large cohort of 731 individuals (SLE/HCs, 357/374) was constructed, which is essential to avoid individual differences for SLE metabolic analysis. In addition, 184 individuals were further classified as an independent cohort for verification, thus ensuring credible diagnostic performance. In contrast, the previous studies of SLE metabolic analysis were conducted based on small numbers of individuals (60‐140 individuals, including 30‐80 SLE and 20‐60 controls),^[^ ^18^, ^19^ ^]^ which often lacked an independent validation cohort and could be easily disturbed by individual difference. Similarly, some pilot studies also found promising results on the specific metabolomic signature in LN patients but might run the risk of overfitting due to the limited sample size (20‐110 individuals).^[^ ^6^, ^20^ ^]^

A high‐performance metabolic detection platform is also critical for SLE diagnosis. The mainstream platforms include NMR spectrometry and MS, besides biochemical/immunoassay.^[^ ^21^ ^]^ For NMR, the spins of nuclei interact with the applied magnetic field to characterize atomic species for untargeted detection,^[^ ^22^ ^]^ but the weak interaction energy involved results in low sensitivity. For comparison, MS affords high‐throughput (≈1000 *m/z* features) and high resolution (± 10 mDa) for both targeted and untargeted metabolic detection.^[^ ^4^, ^23^ ^]^ However, frequently applied MS techniques of GC/LC‐MS call for a considerate experimental time of 0.5–1 h,^[^ ^10a–c^ ^]^ sample volume of 30–50 µL,^[^ ^10c^ ^]^ and prime cost of additional devices and reagents.^[^ ^10^, ^24^ ^]^ Accordingly, nano‐assisted LDI MS we developed is 1) fast without tedious sample pretreatment, due to the surface nano‐crevices of the matrix for in situ size‐selective enrichment of small metabolites rather than large molecules (Figure S1, Supporting Information); 2) of low sample volume (1 µL of serum per individual), due to the unique LDI process in producing efficient cation adduct at low detection limits (8.8–85.4 pmol, Figure S4, Supporting Information); and 3) low‐cost and free of additional devices and reagents, due to the direct recognition of serum microarrays on‐chip (Figure 1A,B) in an antibody‐free manner. Notably, compared to reported nano matrix based on metal oxide,^[^ ^25^ ^]^ the ferric particles can offer high production efficiency and easy‐controlled structure, making it suitable for widespread adoption in large‐scale and clinical testing. Therefore, the ferric particle assisted LDI MS tackled the major challenges in metabolic analysis, serving as a promising detection platform.

A suitable statistical algorithm is essential to interpreting the MS signals for diagnosis due to their complexity (containing ≈120000 data points per SMF). Distinct from the prior metabolomic studies in SLE that adopted the traditional statistical algorithms (eg, principal component analysis (PCA)), sparse learning was applied for SMFs based diagnostic model building towards computer‐aided diagnosis. Sparse learning of the SMFs exhibited a superior diagnostic AUC of 0.950‐0.992 for SLE diagnosis. No performance loss in the independent validation cohort further confirmed the validity of the established diagnostic model (Figure 2 and Figure S6, Supporting Information). The success of sparse learning over other machine learning methods attributes to the sparse regularization and intrinsic sparsity of SMFs (Figure S7 and Table S4, Supporting Information).^[^ ^4a^ ^]^ For sparse regularization, sparse learning allows to gauge the contributions of these *m/z* features via numeric computation and assign high weights to a limited number of biomarkers with relatively high importance. For intrinsic sparsity, only a few *m/z* features are potentially useful to diagnosis, revealed by that only tens of features (66 *m/z* signals) were selected stably with frequency ≥ 95 and statistical significance (*p* < 0.05) as metabolic biomarkers. Therefore, we achieved the advanced sparse learning‐aided diagnosis of SLE based on the SMFs.

In clinical practice, employing the fewer selected metabolites as biomarkers is more practical and feasible than attempting to use the entire set of 66 features.^[^ ^26^ ^]^ We constructed a four‐metabolite panel based on the optimized diagnostic model (66 features), which maintained the diagnostic performance of the whole profiling of SMFs. The panel was further validated in a small cohort of SLE patients versus RA patients. The four metabolites (imidazoleacetic acid, 2‐hydroxyadipic acid, glucose, and pseudouridine) identified in the current study showed certain consistency with previous metabolic profiling or pathogenesis studies and revealed novel discoveries. Glucose was found to be increased in SLE patients by several different metabolic profiling studies.^[^ ^20^, ^27^ ^]^ The alternation of glucose could be raised by the mitochondrial dysfunction and consequent energy abnormality of SLE patients, agreeing with CD4^+^ T cells metabolism.^[^ ^28^ ^]^ 2‐Hydroxyadipic acid, an aliphatic acyclic compound, is another biomarker associated with the energy abnormality, which is involved in fatty acid metabolism.^[^ ^29^ ^]^ Clinically, our group has reported that metformin, originally a medication for diabetes, reduced frequency of major flares in SLE patients,^[^ ^30^ ^]^ indicating that the metabolism of glucose and energy is a promising therapeutic target in SLE. In addition to energy metabolism, the disorder of histamine metabolism was also found to be associated with SLE in the present study, which is mainly involved in immune regulation and allergy.^[^ ^31^ ^]^ Imidazoleacetic acid is the oxidative product of histamine,^[^ ^32^ ^]^ an important immunomodulator that regulates allergic inflammatory reactions and other physiological processes.^[^ ^31^, ^33^ ^]^ The abnormality of histamine metabolism in SLE patients has been reported in previous work,^[^ ^18^, ^34^ ^]^ indicating histamine metabolism could be a key factor in the pathogenesis of SLE and other immune diseases. Another interesting biomarker is pseudouridine, also known as 5‐ribosyluracil, associated with the nucleoside metabolism. Previous metabolic profiling studies haven't suggested the abnormality of nucleoside metabolism of SLE, which, however, has been reported to be related to other autoimmune diseases, such as Graves’ disease and Aicardi‐Goutières syndrome.^[^ ^35^ ^]^ The abnormality of nucleoside metabolism could indicate the dysfunctions for activation and proliferation of immune cells, which demand more complex and energy expensive nucleotide synthesis pathway.^[^ ^36^ ^]^ Meanwhile, nucleotide metabolism is involved in the regulation of mtDNA‐dependent innate immunity.^[^ ^37^ ^]^ Our finding suggested that nucleotide metabolism has the potential as a novel target for therapeutic interventions to prevent SLE. Although the biomarker panel identified in this study differed from previous reports, the pathways and biological roles of biomarkers were generally consistent across the present and previous studies, which were mainly involved in the inflammation responses, mitochondrial dysfunction, carbohydrate and lipid metabolism abnormality.^[^ ^18^, ^34^, ^38^ ^]^ The different metabolites within the same metabolic pathways were identified as biomarkers across the present and prior studies could be due to different ionization sources. The mass spectrometers with different ionization sources generally produce distinct SMFs in metabolic analysis, which can yield a particular machine learning model, resulting in the difference of high contribution biomarkers. On the other hand, in the present study, the purpose of the construction of biomarker panel is to efficiently identify SLE patients with the use of as few biomarkers as possible, which caused that *m/z* features of some differential metabolites were not selected to the biomarker panel and identified. In clinical practice, employing the 4 selected metabolites as biomarkers is more practical and feasible than attempting to use the entire set of 66 features. These 4 metabolites have demonstrated substantial diagnostic performance and can be readily utilized for diagnostic purposes in a clinical setting. They offer a balance between accuracy and clinical applicability, making them suitable for translation into clinical practice.

## 5 Conclusion

We acquired the SMFs of 915 individuals by nano‐assisted LDI MS and achieved the SLE diagnosis with AUC of 0.950–0.992 by sparse learning of the SMFs. We preliminarily constructed a biomarker panel of 4 metabolites for SLE patients, demonstrating their unique metabolic pattern compared to HCs and RA patients. In parallel to recent genomic and proteomic advances, this work would inspire the pathogenic insights of SLE emerging in metabolomics and shed light on the clinical tool for precision diagnosis and monitoring in the near future.

There are still several limitations and future research lines to be stated, including that 1) MS system is required to record the SMFs and may hinder its potential application in point‐of‐care (POC) testing; 2) There are opportunities for enhancing cohort collection, including improving the matching between patient and healthy groups, conducting screenings for untreated patients, incorporating disease control groups (such as Sjogren's syndrome), and gathering samples from diverse ethnic backgrounds. These efforts will aid in achieving a more comprehensive understanding of the mechanisms underlying SLE; 4) a combination of multi‐modal information would enhance the outreach and applicability of our approach.

## Conflict of Interest

The authors have filed patents using the nano‐assisted LDI MS methods to detect and diagnose SLE patients.

## Supporting information

Supporting Information

## Data Availability

The data that support the findings of this study are available from the corresponding author upon reasonable request.
