# Supplementary material for: Serum Metabolic Fingerprints Characterize Systemic Lupus Erythematosus
Source: Adv Sci (Weinh). 2023 Nov 12;11(2):2304610. doi: 10.1002/advs.202304610 (PMC10787061; doi:10.1002/advs.202304610)
Supplement: Supplementary file 1 — Supporting Information [file ADVS-11-2304610-s001.pdf]

ADVANCED SCIENCE
Open Access

Supporting Information

for *Adv. Sci.*, DOI 10.1002/advs.202304610

Serum Metabolic Fingerprints Characterize Systemic Lupus Erythematosus

Shunxiang Li, Huihua Ding, Ziheng Qi, Jing Yang, Jingyi Huang, Lin Huang, Mengji Zhang, Yuanjia Tang, Nan Shen, Kun Qian, Qiang Guo* and Jingjing Wan**

Supplementary Information

Serum metabolic fingerprints characterize systemic lupus erythematosus

Shunxiang Li, Huihua Ding, Ziheng Qi, Jing Yang, Jingyi Huang, Lin Huang, Mengji Zhang, Yuanjia Tang, Nan Shen, Kun Qian, Qiang Guo,* and Jingjing Wan**

1. Methods

1.1. Chemicals and reagents

Ferric chloride (97%), trisodium citrate (99%), sodium acetate (99%), ethylene glycol (99.5%), citric acid monohydrate (99.5%), absolute ethanol, potassium chloride (99.5%), and sodium chloride (99.5%) were purchased from Sinopharm Chemical Reagent Beijing Co. Ltd. (Beijing, China). Trifluoroacetic acid (TFA, 99.5%) was purchased from Macklin Biochemical Co., Ltd. (Shanghai, China). D-glucose (99.5%), L-valine (98%), D-mannitol (99%), L-proline, L-arginine (99.5%), creatinine, α-cyano-4-hydroxycinnamic acid (CHCA, 99%), albumin from bovine serum (BSA, 98%), and acetonitrile (ACN, 99%) were obtained from Sigma-Aldrich (St. Louis, MO, USA). All aqueous solutions were prepared using deionized water (18.2 MΩ cm, Milli-Q, Millipore, GmbH) throughout the experiments.

1.2. Preparation and characterization of materials

The ferric particles were prepared using a solvothermal method according to the literatures.[1] Dynamic light scattering (DLS) and zeta potential were performed by dispersing the ferric particles in water at 25°C, utilizing Nano-ZS90 instrument (Malvern, Worcestershire, UK). Scanning electron microscopy (SEM) images were recorded on Hitachi S-4800 (Hitachi, Japan) by placing a drop of material suspension on the aluminum foil. Transmission electron microscopy (TEM) image, high-resolution TEM (HR TEM) image, elemental mapping image, and selected area electron

diffraction (SAED) image were collected using a JEOL JEM-2100F instrument, by depositing 10 μL of material suspension onto a copper grid before observation. Ultraviolet-visible (UV-Vis) spectrum was obtained using UV1900 spectrophotometer (AuCy, China), by dispersing material in deionized water at room temperature. The digital photo was taken by Panasonic DC-GF10K.

1.3. Cohort characteristics and serum harvesting

A total of 915 serum samples from systemic lupus erythematosus (SLE) patients and healthy controls (HCs) were enrolled in this study, which were randomly divided into the discovery cohort and validation cohort (Table S1 and S2). There were 731 individuals in the discovery cohort (including 357 SLE patients and 374 healthy controls (HCs)) and 184 individuals in the validation cohort (including 91 SLE patients and 93 HCs). No significant difference in age and sex was observed between SLE patients and HCs ($p > 0.05$) in the discovery cohort and validation cohort. Notably, the healthy volunteers, who showed no signs of arthralgia, heart failure, renal failure, autoimmune disease, other inflammatory conditions, and major diseases, were enrolled as HCs. All SLE patients were diagnosed according to 2012 Systemic Lupus International Collaborating Clinics (SLICC).[2] The organ involvements were verified according to the pathological examinations and medical history. Among the SLE patients, 6 major organ involvements were investigated, including 228 cases of patients with renal involvement (186/42, discovery/validation), 203 (159/44, discovery/validation) with mucocutaneous involvement, 134 (106/28, discovery/validation) with hematological involvement, 123 (94/19, discovery/validation) with musculoskeletal involvement, and 87 (71/16, discovery/validation) with cardiorespiratory involvement. In addition, the other 2 SLE organ involvements were of limited sample size (neuropsychiatric of 19 cases and gastrointestinal of 25 cases).

Independently, a cohort of 27 rheumatoid arthritis (RA) patients with corresponding serum samples were also included for verifying the efficiency of the metabolic biomarker panel (Table S12). All the 27 RA patients were diagnosed according to 2010 American College of Rheumatology/European League Against Rheumatism (ACR/EULAR) rheumatoid arthritis classification criteria.[3] For excluding the potential bias, a small cohort of 27 SLE patients was selected from above 357 SLE patients by propensity score matching (PSM) for matching the age, gender, and medication (prednisone, methotrexate, leflunomide, and hydroxychloroquine) of RA patients (Table S15), which were proved by statistical examinations. From the above cohort of SLE and RA (357/27), 20 patients (SLE/RA, 10/10) with naïve treatment were also selected to further exclude the potential interference on serum metabolic fingerprints (SMFs) by medication treatment (Table S15).

All serum samples of above individuals were collected from the Department of Rheumatology, Renji Hospital. The serum samples of SLE patients were collected from the admitted patients, and HCs were collected from the outpatient clinic. All the serum samples were collected after 8-10 hours of overnight fasting. Specifically, the venous whole blood was collected from each participant into 5 mL vacutainer tubes. After 1-1.5 hours of incubation at room temperature, the tubes were centrifuged for 10 min at a speed of 2000 rpm/450 G to obtain serum sample. The supernatant was separated carefully and aliquoted into 500 μL and stored at -80°C before use. All the investigation protocols in this study were in accordance with the Declaration of Helsinki and approved by the institutional ethics committees of the Renji Hospital, Shanghai Jiao Tong University (RA-2019-156).

1.4. MS analysis

The serum samples, small molecules, and prepared mixtures were detected by LDI MS, using ferric particles, CHCA, and blank control as matrices, respectively. For the matrix

preparation, ferric particles were dispersed in deionized water with 1 mg mL^{-1}. The CHCA was prepared by dissolving in a 0.1% TFA buffer (water/ACN, 50/50, v/v) with a concentration of 5 mg mL^{-1}. For a typical LDI MS analysis, 1 μL of serum/standard solution of the small molecule was dropped and mixed with 1 μL matrix suspension of ferric particles/CHCA/blank control. For examining the salt tolerance and protein endurance, a high concentration of BSA (5 mg mL^{-1}) or salt (NaCl, 0.5 M) was respectively mixed with standard small molecules (arginine, glucose, and mannitol) of 1 mg mL^{-1}. Mass spectra were collected in the reflection mode employing delayed extraction on Autoflex (Bruker Daltonics GmbH, Germany) with the Nd:YAG laser of 355 nm, a frequency of 500 Hz, and total shots of 2000. Mass calibration was conducted using standard molecules for accurate mass measurement (± 10 mDa) of both Na^+ adducted ($[M + Na]^+$) and K^+ adducted ($[M + K]^+$) signals. Five independent experiments were performed for each sample to exclude the accidental error.

The LC-ESI-$HRMS^2$ analysis (Thermo Q Exactive HF) was performed for identification and validation of the high-contribution mass to charge (*m/z)* features within LDI-MS SMFs. For preparation of each serum sample, 200 μL of methanol/acetonitrile (50/50, v/v) monophasic mixture was added to 50 μL of serum sample and placed at -20 °C for 2 hours. Next, the mixture was centrifuged at 13000 g for 20 minutes. The upper layer solution was then dried by centrifugation and re-dissolved in 100 μL of methanol/water (30/70, v/v). The quality control (QC) sample was prepared by mixing 5 μL of each sample.

For LC-MS/MS metabolic analysis, both HILIC and RP-C18 separation modes were utilized in positive and negative electrospray ionization modes. The Welch Ultimate AQ-C18 column (particle size, 5μm; 250mm (length) × 2.1 mm (i.d.)) and SeQuant ZIC-HILIC column (particle size, 5μm; 150mm (length) × 2.1 mm (i.d.)) were used in the experiment. The column was maintained at 35 °C. The flow rate was set at 0.2 mL min^{-1} and the sample injection volume was 5 μL. For RP-C18 separation, a gradient

mobile phase system was used consisting of mobile phase A (0.1% formic acid in water) and B (0.1% formic acid in acetonitrile): 0-15 min: 50% B; 15-16 min: 50% B to 60% B; 16-18 min: 60% B to 90% B; 18-20 min: 90% B; 20-21 min: 90% B to 0% B; 21-30 min: 0% B. For HILIC separation, the mobile phase was 0.2% NH_4OH and 5mM NH_4HCO_2 in water for A and pure acetonitrile for B. The gradient elution was set as follow: 0-18 min: 90% B to 40% B; 18-20min: 40% B; 20-21 min: 40% B to 90% B; 21-30 min: 90% B. The QC sample was analyzed every ten injections to monitor the data quality and be used for data normalization. For data acquisition, data-dependent acquisition mode was adapted. The MS scan time was 50 ms/scan and the resolution was 60,000. The MS/MS scan time was 50 ms/scan and the resolution was 15,000. Top 10 most intense ions were selected for MS/MS fragmentation (30 NCE).

1.5. Machine learning algorithms

Six machine learning methods were applied for identifying the SLE patients from HCs, including sparse learning,[4] decision tree,[5] logistic regression,[6] supporting vector machine (SVM),[7] K-nearst neighbors (kNN) ,[8] and random forest.[9] Sparse learning was a combination of least absolute shrinkage and selection operator (LASSO) and ridge regression, and was subjected to the formula below:

$$\widehat{\boldsymbol{\beta}}(\boldsymbol{\lambda_1}, \boldsymbol{\lambda_2}) = \arg\min_{\beta} \left(\frac{||Y - X\beta||_2^2}{2n} + \lambda_1 ||\beta||_1 + \frac{\lambda_2}{2} ||\beta||_2^2 \right) \quad (1)$$

where λ_1 and λ_2 were parameters employed for MS data processing.

In detail, the sparse learning was conducted by 5-fold cross-validation for 20 rounds and obtained 100 diagnostic models in total. The diagnostic performance was evaluated by area-under-the-curve (AUC) of receiver operation curve (ROC). The SMFs were acquired by preprocessing the raw MS spectra, including baseline correction, peak detection, extraction, alignment, normalization, and standardization utilizing MATLAB (R2016a, The Mathworks, Natick, MA). And the mean spectrum of five independent mass spectra of each sample was used to construct SMFs towards diagnostic use.

1.6. Construction of metabolic biomarker panel

The main steps for constructing the metabolic biomarker panel included the following 4 steps. Firstly, the raw mass spectra of both SLE patients and HCs were obtained by nano-assisted LDI MS detection, containing ~ 120,000 data points. Then, the sparse learning was applied for treating the massive data of raw mass spectra, yielding the SMFs with 908 *m/z* features for each serum sample. The specific *m/z* features were selected from the SMFs according to their performances in SLE diagnostic model, including selection frequency (≥ 95) and statistical significance ($p < 0.05$). Finally, the identification and validation of the specific *m/z* features within LDI-MS SMFs were conducted according the LC-MS/MS data from an independent validation cohort. Specifically, for each specific *m/z* feature, the corresponding *m/z* feature within LC-MS/MS was determined according to the molecular mass and fold changes of intensities, which can be regarded as the signal of the same compound. Next, through the corresponding LC-MS/MS data, the specific features were annotated to metabolites via accurate mass and MS/MS matching with the human metabolome database (https://hmdb.ca).

1.7. Statistical analysis

The χ^2 test and Mann-Whitney test were respectively conducted for examining if there were significant differences in the sex and age between SLE patients and HCs (Table S1 and S2). The two-tailed t-test was conducted to examine the diagnostic differences between sparse learning and other machine learning methods in AUC ($p < 0.0001$ in discovery cohort and $p = 0.0002$ in validation cohort), sensitivity ($p = 0.0064$ in discovery cohort and $p = 0.0081$ in validation cohort), and specificity ($p = 0.0009$ in discovery cohort and $p = 0.0201$ in validation cohort). The two-tailed t-test was also conducted to verify the differences of diagnostic performances afforded by SMFs and

the major SLE organ involvements, demonstrating the superiority of SMFs over organ involvements ($p < 0.0001$/$p < 0.0001$ in specificity and p of 0.0079/0.0048 in AUC, respectively in discovery and validation cohort). The two-tailed t-test was applied for examining the significant differences of the potential metabolic biomarkers of SLE patients from both HCs and RA patients (Table S6 and S14). Moreover, principal component analysis, an unsupervised clustering method, was utilized for validating the capability of a metabolic biomarker panel in SLE identification from RA patients. To exclude the drug effect, the Propensity score matching (PSM) method was applied for selecting SLE patients to match with RA patients. The power analysis was conducted by Metaboanalyst (http://www.metaboanalyst.ca/) to study the minimal sample size for building a robust diagnostic model.[10] The fold change analysis was conducted to investigate the variation trends of SLE biomarkers compared to HCs and RA patients.

References

[1] a) V. Vedarethinam, L. Huang, W. Xu, R. Zhang, D. D. Gurav, X. Sun, J. Yang, R. Chen, K. Qian, *Small* **2019**, *15* (3), 1970020; b) J. Wu, X. Wei, J. R. Gan, L. Huang, T. Shen, J. T. Lou, B. H. Liu, J. X. J. Zhang, K. Qian, *Adv. Funct. Mater.* **2016**, *26* (22), 4016.

[2] M. Petri, A. M. Orbai, G. S. Alarcon, C. Gordon, J. T. Merrill, P. R. Fortin, I. N. Bruce, D. Isenberg, D. J. Wallace, O. Nived, G. Sturfelt, R. Ramsey-Goldman, S. C. Bae, J. G. Hanly, J. Sanchez-Guerrero, A. Clarke, C. Aranow, S. Manzi, M. Urowitz, D. Gladman, K. Kalunian, M. Costner, V. P. Werth, A. Zoma, S. Bernatsky, G. Ruiz-Irastorza, M. A. Khamashta, S. Jacobsen, J. P. Buyon, P. Maddison, M. A. Dooley, R. F. van Vollenhoven, E. Ginzler, T. Stoll, C. Peschken, J. L. Jorizzo, J. P. Callen, S. S. Lim, B. J. Fessler, M. Inanc, D. L. Kamen, A. Rahman, K. Steinsson, A. G. Franks, L. Sigler, S. Hameed, H. Fang, N. Pham, R. Brey, M. H. Weisman, G. McGwin, L. S. Magder, *Arthritis Rheumatol.* **2012**, *64* (8), 2677.

[3] D. Aletaha, T. Neogi, A. J. Silman, J. Funovits, D. T. Felson, C. O. Bingham, N. S. Birnbaum, G. R. Burmester, V. P. Bykerk, M. D. Cohen, B. Combe, K. H. Costenbader, M. Dougados, P. Emery, G. Ferraccioli, J. M. W. Hazes, K. Hobbs, T. W. J. Huizinga, A. Kavanaugh, J. Kay, T. K. Kvien, T. Laing, P. Mease, H. A. Menard, L. W. Moreland, R. L. Naden, T. Pincus, J. S. Smolen, E. Stanislawska-Biernat, D. Symmons, P. P. Tak, K. S. Upchurch, J. Vencovsky, F. Wolfe, G. Hawker, *Ann. Rheum. Dis.* **2010**, *69* (9), 1580.

[4] S. Ryali, T. Chen, K. Supekar, V. Menon, *Neuroimage* **2012**, *59* (4), 3852.

[5] J. R. Quinlan, *Mach. Learn. (Netherlands)* **1986**, *1* (1), 81.

[6] S. Nusinovici, Y. C. Tham, M. Y. C. Yan, D. S. W. Ting, J. L. Li, C. Sabanayagam, T. Y. Wong, C. Y. Cheng, *J. Clin. Epidemiol.* **2020**, *122*, 56.

[7] C. J. C. Burges, *Data Min. Knowl. Discov.* **1998**, *2* (2), 121.

[8] K. Q. Weinberger, L. K. Saul, *J. Mach. Learn. Res.* **2009**, *10*, 207.

[9] L. Breiman, *Mach. Learn. (Netherlands)* **2001**, *45* (1), 5.

[10] a) C. R. Molins, L. V. Ashton, G. P. Wormser, B. G. Andre, A. M. Hess, M. J. Delorey, M. A. Pilgard, B. J. Johnson, K. Webb, M. N. Islam, A. Pegalajar-Jurado, I. Molla, M. W. Jewett, J. T. Belisle, *Sci. Transl. Med.* **2017**, *9* (403), 12; b) X. X. Wang, K. L. Yang, Q. L. Wu, L. J. Y. Kim, A. R. Morton, R. C. Gimple, B. C. Prager, Y. Shi, W. C. Zhou, S. Bhargava, Z. Zhu, L. Jiang, W. W. Tao, Z. X. Qiu, L. J. Zhao, G. X. Zhang, X. Q. Li, S. Agnihotri, P. S. Mischel, S. C. Mack, S. D. Bao, J. N. Rich, *Sci. Transl. Med.* **2019**, *11* (504), 14.

[11] X. Ouyang, Y. Dai, J. L. Wen, L. X. Wang, *Lupus* **2011**, *20* (13), 1411.

[12] T. Wu, C. Xie, J. Han, Y. Ye, J. Weiel, Q. Li, I. Blanco, C. Ahn, N. Olsen, C. Putterman, R. Saxena, C. Mohan, *PLoS One* **2012**, *7* (6), e37210.

[13] A. A. Bengtsson, J. Trygg, D. M. Wuttge, G. Sturfelt, E. Theander, M. Donten, T. Moritz, C.-J. Sennbro, F. Torell, C. Lood, I. Surowiec, S. Rannar, T. Lundstedt, *PLoS One* **2016**, *11* (7).

[14] A. Guleria, A. Pratap, D. Dubey, A. Rawat, S. Chaurasia, E. Sukesh, S. Phatak, S. Ajmani, U. Kumar, C. L. Khetrapal, P. Bacon, R. Misra, D. Kumar, *Sci. Rep.* **2016**, *6* (1), 35309.

[15] B. Yan, J. Huang, C. M. Zhang, X. Hu, M. Gao, A. X. Shi, W. B. Zha, L. Y. Shi, C. B. Huang, L. P. Yang, *Mod. Rheumatol.* **2016**, *26* (6), 914.

[16] J. Li, X. W. Xie, H. Zhou, B. Wang, M. J. Zhang, F. Y. Tang, *Lupus* **2017**, *26* (11), 1166.

[17] A. Guleria, S. Phatak, D. Dubey, S. Kumar, A. Zanwar, S. Chaurasia, U. Kumar, R. Gupta, A. Aggarwal, D. Kumar, R. Misra, *J. Proteome Res.* **2018**, *17* (7), 2440.

[18] Y. Li, L. Liang, X. Deng, L. Zhong, *Int. J. Clin. Exp. Patho.* **2019**, *12* (3), 857.

[19] Q. Zhang, X. Li, X. Yin, H. Wang, C. Fu, H. Wang, K. Li, Y. Li, X. Zhang, H. Liang, K. Li, H. Li, Y. Qiu, *Rheumatology* **2021**, *60* (2), 598.

2. Figures

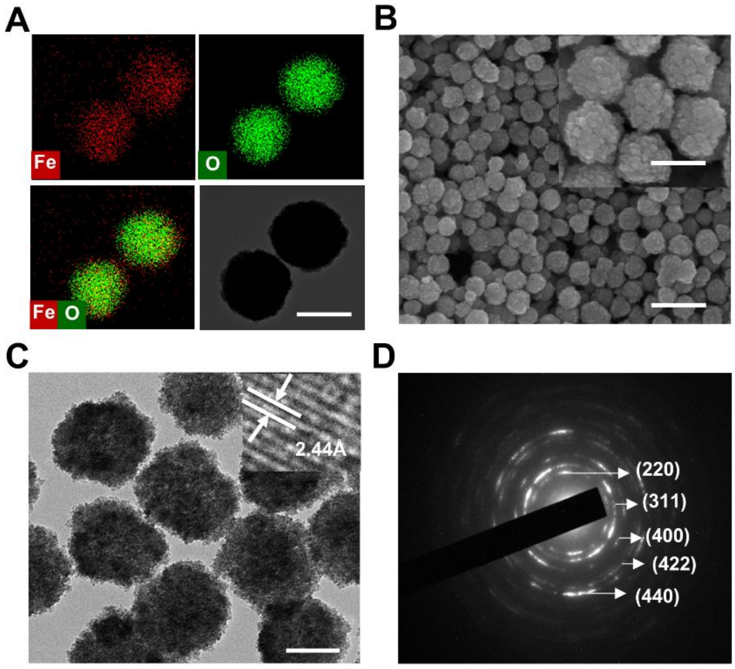

Figure S1. Characterization of ferric particles. (A) Elemental mapping of ferric nanoparticles with Fe in red, O in green, overlapped (Fe + O), and initial transmission electron microscopy (TEM) image. (B) Scanning electron microscopy (SEM) image and its inset displayed the rough surface of ferric nanoparticles. (C) TEM image of ferric particles with high-resolution TEM (HR TEM) as inset. The HR TEM image demonstrated the lattice of 2.44 Å, referring to the crystalline plane of (311) for ferric particles. The scale bar in (A), (B), inset of (B), and (C) is 200 nm/500 nm/200 nm/100 nm. (D) Selected area electron diffraction (SAED) image displays the typical rings of (220), (311), (400), (422), and (440) for ferric nanoparticles.

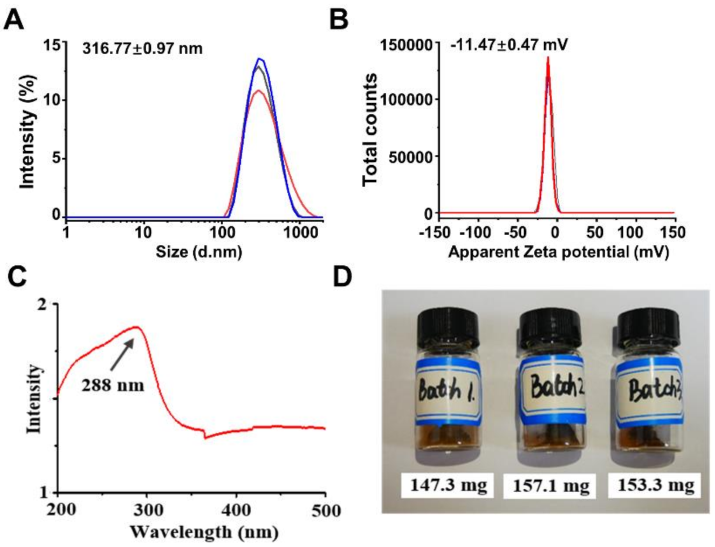

Figure S2. Size distribution, zeta potential, ultraviolet-visible (UV-vis) spectrum, and weight measurement of ferric nanoparticles. Typical (A) size and (B) Zeta potential distribution of ferric nanoparticles based on the three independent measurements. The averaged value with standard deviation (SD) is labeled in (A) and (B). (C) UV-vis spectrum of ferric nanoparticles showed an absorbance peak at 288 nm. (D) The weight of three independent batches of ferric nanoparticles.

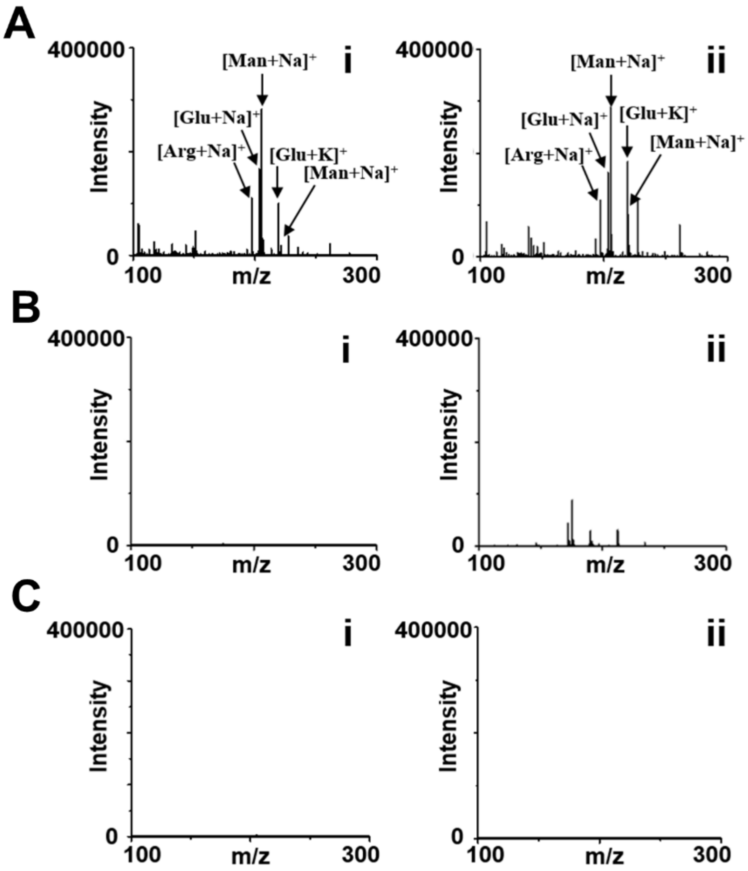

Figure S3. Evaluation of protein endurance and salt tolerance of different matrices. Typical MS spectra by using the matrix of (A) ferric nanoparticles, (B) α-cyano-4-hydroxycinnamic acid (CHCA), and (C) blank control for examining the (i) protein tolerance and (ii) salt tolerance. For protein tolerance, the mixture of the BSA (5 mg mL^{-1}) with 1 mg mL^{-1} of arginine (Arg), glucose (Glu), and mannitol (Man) was detected. For salt tolerance, the mixture of NaCl solution (0.5 M) with 1 mg mL^{-1} of arginine (Arg), glucose (Glu), and mannitol (Man) was detected. Adducts of Na^{+} and K^{+} are labeled in (A) and (B). The laser desorption/ionization mass spectrometry (LDI MS) detection was conducted in positive ion mode.

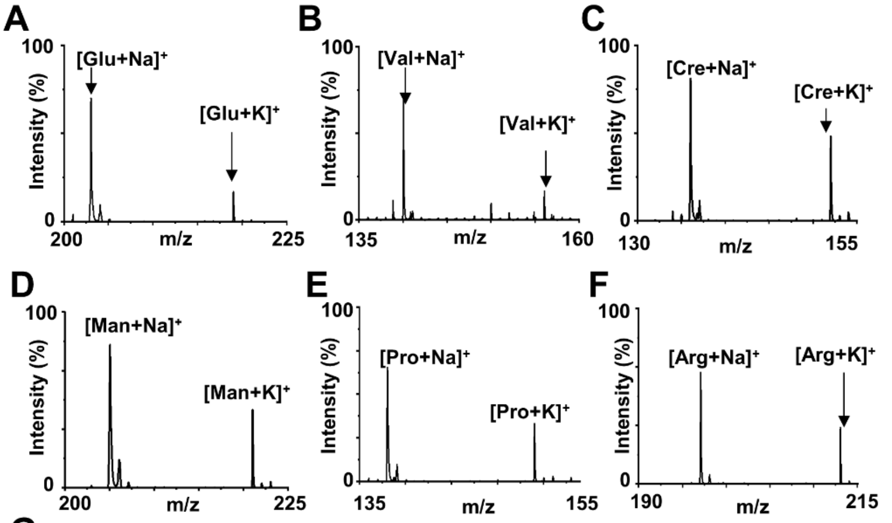

G

Metabolite	Glucose	Valine	Creatinine	Mannitol	Proline	Arginine
Detection limit (pmol)	55.5	85.4	8.8	54.9	8.7	57.4

Figure S4. Mass spectra of typical small molecule metabolites. Small molecule metabolites were of 100 μg mL^{-1}, including (A) glucose (Glu), (B) valine (Val), (C) creatinine (Cre), (D) mannitol (Man), (E) proline (Pro), and (F) arginine (Arg). Detection limits of small molecules are summarized in (G). All the LDI MS experiments were performed in positive ion mode.

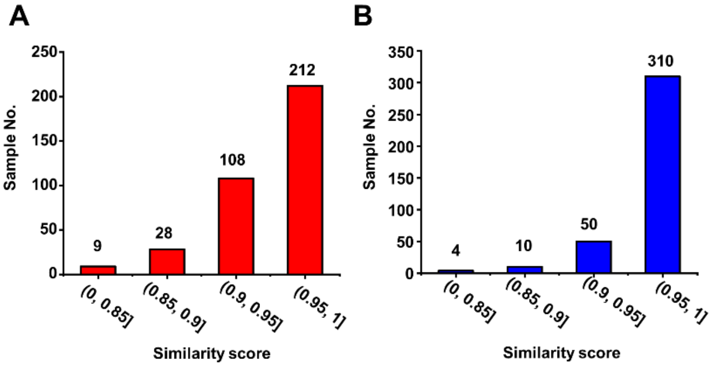

Figure S5. The frequency distribution of similarity scores based on the cosine correlation method for (A) 357 systemic lupus erythematosus (SLE) patients and (B) 374 healthy controls (HCs). The similarity scores of > 0.9 accounted for 89.6% of SLE patients and 96.3% of HCs, respectively, illustrating the limited intra-group difference.

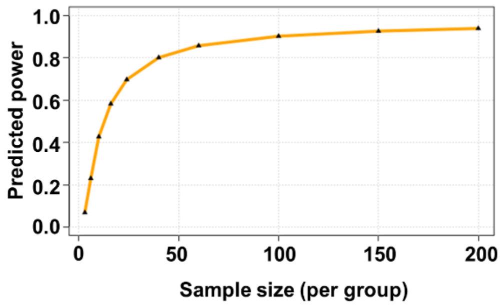

Figure S6. The power analysis of pilot data. The serum metabolic fingerprints (SMFs) of 20 samples (SLE/HC, 10/10) were utilized for studying the diagnostic performance with the variable sample numbers. The predicted power of ≥ 0.8 can be achieved by a sample size of ≥ 40 for each group.

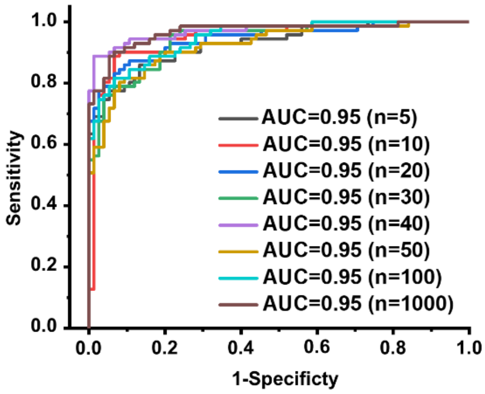

Figure S7. The receiver operating characteristics (ROC) curves for SLE identification from HCs. The diagnostic model number (n) of sparse learning was adjusted from 5 to 1000 with the corresponding area-under-the-curve (AUC) labeled. As the results displayed, the diagnostic performance of sparse learning was stable as tunning the model number.

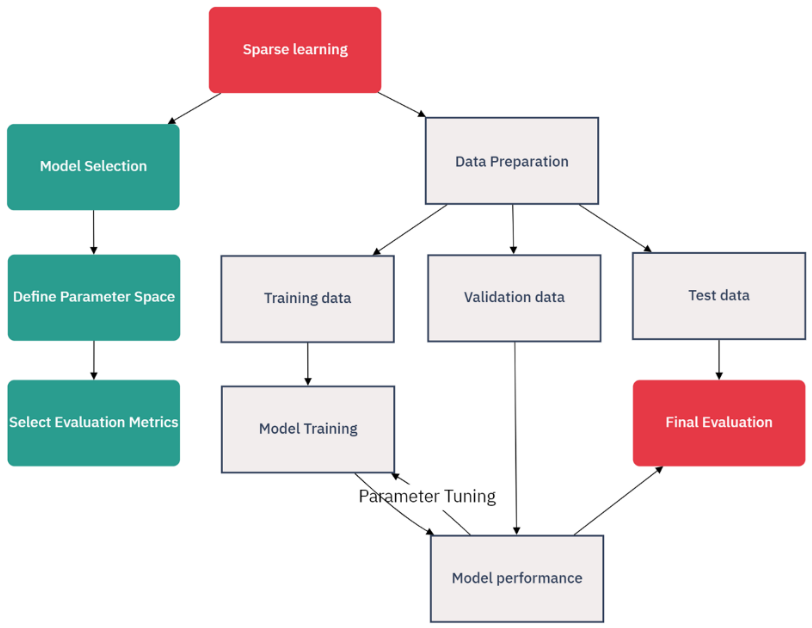

Figure S8. The flowchart for the process of parameter optimization for sparse learning. The diagnostic performance was evaluated by area-under-the-curve (AUC) of receiver operation curve (ROC). Data preprocessing includes: baseline correction, peak detection, extraction, alignment, normalization, and standardization. The data preprocessing divides all the data into three groups. The training data is used for model training to obtain model parameter data, which is subsequently validated with the validation data to calculate the AUC value. This process is iteratively repeated to identify the model parameters that achieve the highest AUC value. These parameters are ultimately evaluated and confirmed using the test data.

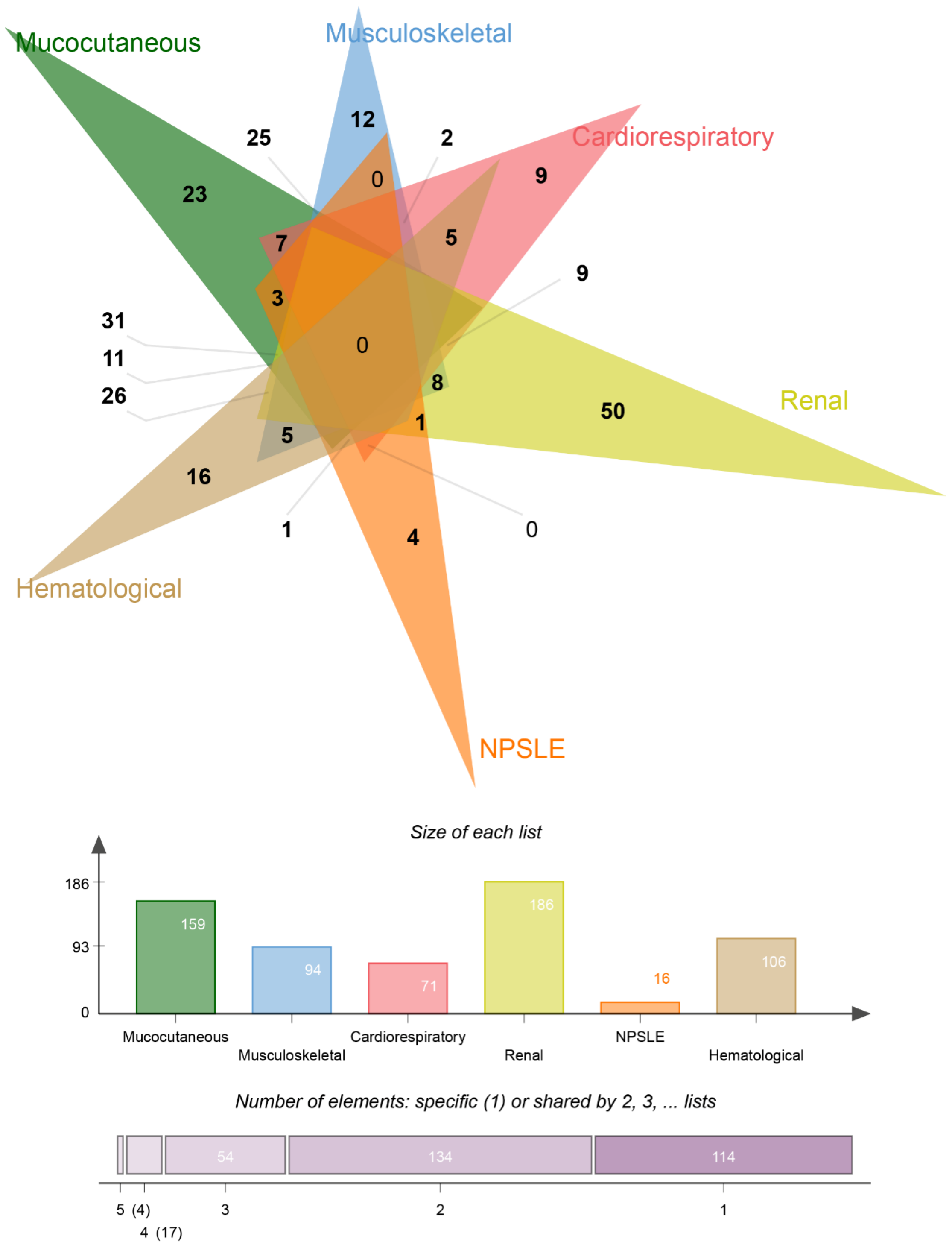

Figure S9. The organ involvement status of SLE discovery cohort. Venn diagrams can illustrate whether patients have single-organ involvement or multiple-organ involvement, with the majority of SLE patients experiencing simultaneous effects on multiple organs.

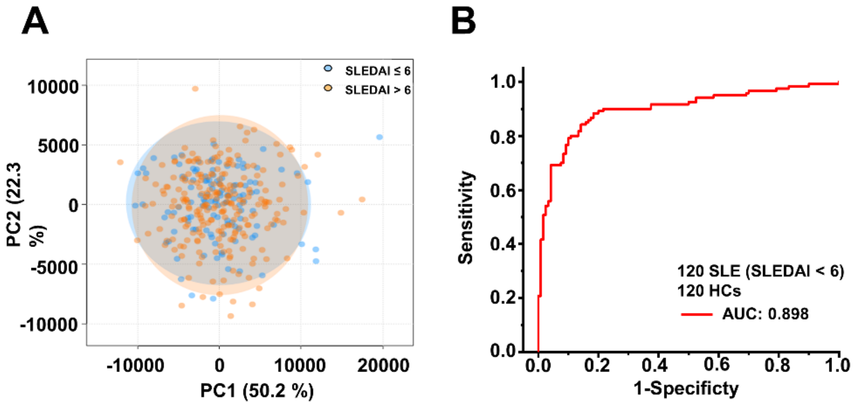

Figure S10. The investigation of disease activity of SLE patients with corresponding SMFs. (A) The SMFs based principal component analysis (PCA) of 237 SLE patients with high disease activity (SLEDAI > 6) and 120 SLE patients with low disease activity (SLEDAI ≤ 6) in discovery cohort. (B) The ROC curves for identifying 120 SLE patients with low disease activity (SLEDAI≤ 6) from HCs in discovery cohort.

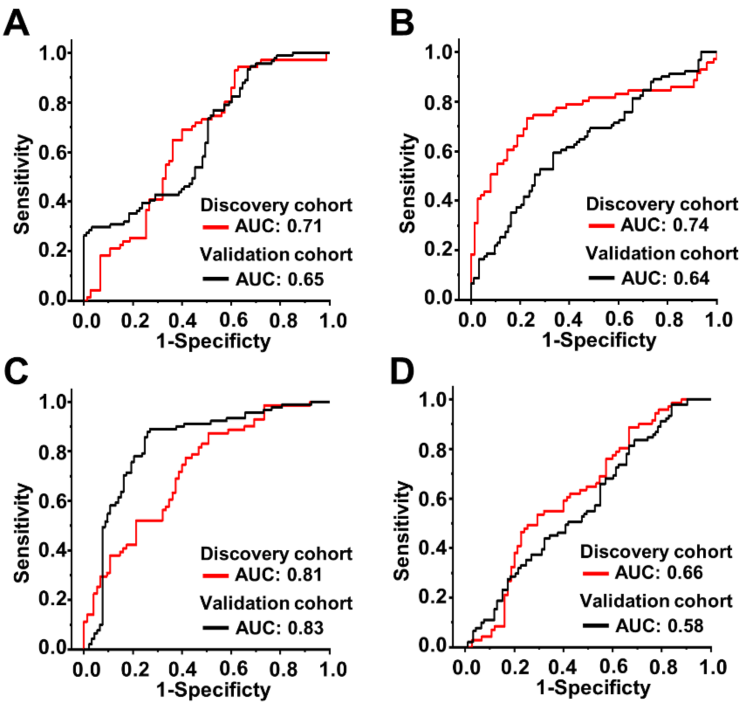

Figure S11. The ROC curves for SLE identification from HCs. ROC curve by sparse learning of (A) imidazoleacetic acid, (B) 2-hydroxyadipic acid, (C) glucose, and (D) pseudouridine, in the discovery cohort (SLE/HC, 357/374, in red line) and validation cohort (SLE/HC, 91/93, in black line). AUC is respectively labeled.

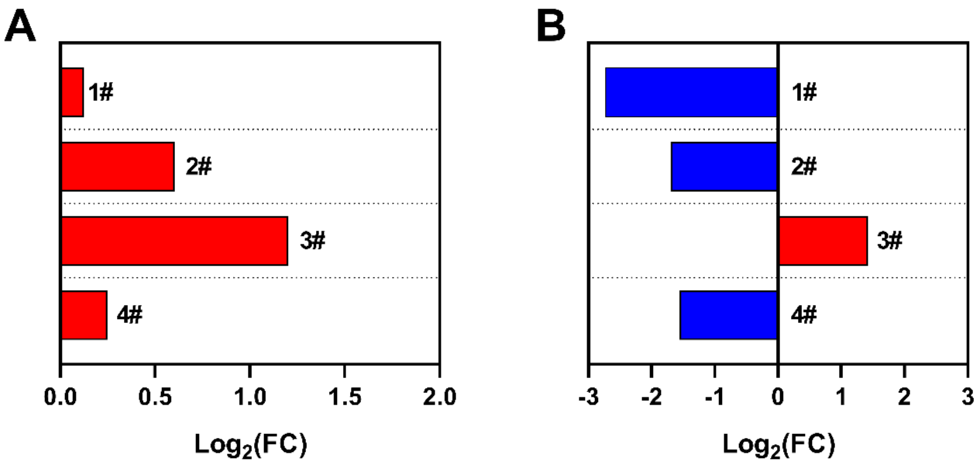

Figure S12. The fold change (FC) plot. (A) The FC plot of four potential biomarkers by comparing the SLE patients with HCs. (B) The FC plot of four potential biomarkers by comparing the SLE patients with rheumatoid arthritis (RA) patients.

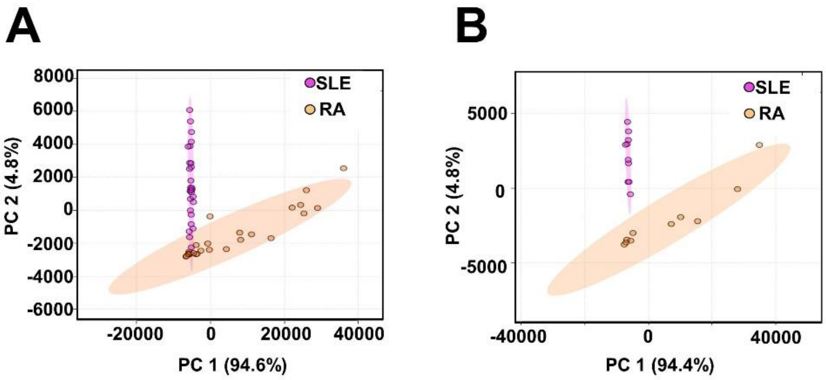

Figure S13. Identification of SLE patients from RA patients based on the metabolic biomarker panel. (A) The scores plot of 27 SLE patients and 27 RA patients in Table S15 by principal component analysis (PCA). (B) The scores plot of 20 patients (SLE/RA, 10/10) with naïve treatment by PCA.

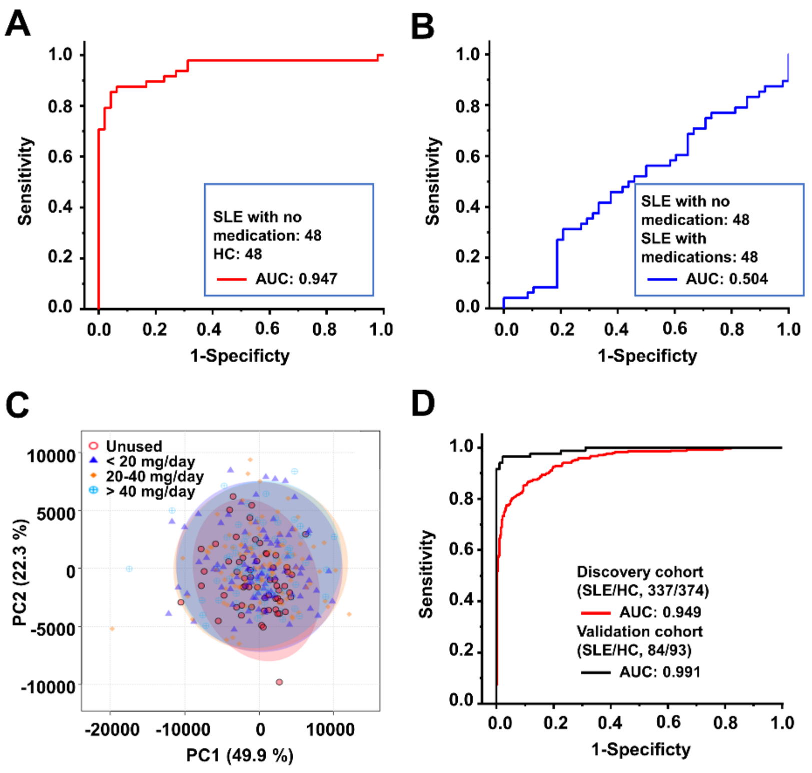

Figure S14. The medication and complication influence in identifying the SLE patients from HCs. (A) The ROC curve for identification of 48 SLE patients who received no medication with 48 HCs. (B) The ROC curve for identifying the 48 SLE patients who received no medication with 48 HCs from 48 SLE patients who received medication. The sample cohort in (A) and (B) used for diagnostic model building was displayed in Table S16. (C) The PCA analysis of SLE patients who received the different dosages of corticosteroids, including 15 individuals who received no corticosteroids, 100 individuals with < 20 mg day^{-1}, 104 individuals with 20-40 mg day^{-1}, and 79 individuals with > 40 mg day^{-1} in the discovery cohort of Table S2. No clear cluster was formed based on the corticosteroid dosage, illustrating its minor role in the SLE diagnostic model. (D) The ROC curves of discovery cohort and validation cohort after excluding SLE patients with complications of diabetes and dyslipidemia.

3. Tables

Table S1. Demographic information of discovery cohort and validation cohort.

**Cohort**	**Characteristics**	**SLE (n = 357)**	**HCs (n = 374)**	***P***
Discovery cohort	Number, N	357	374	
	Age (yr), mean/median	38/36	39/39	0.244 [a]
	Gender, N (%)			
	Female	334 (93.6%)	348 (93.0%)	0.783 [b]
	Male	23 (6.4%)	26 (7.0%)	
	BMI, mean/median	21.6/21.5		
	Diabetes, N	13	0	
	Dyslipidemia, N	7	0	
Validation cohort	Number, N	91	93	
	Age (yr), mean/median	38/35	39/39	0.318 [a]
	Gender, N (%)			
	Female	88 (96.7%)	84 (90.3%)	0.080 [b]
	Male	3 (3.3%)	9 (9.7%)	
	BMI, mean/median	21.8/20.6		
	Diabetes, N	7	0	
	Dyslipidemia, N	0	0	

[a] is calculated by the Mann-Whitney U test.

[b] is calculated by χ^2 test.

Table S2. Clinical features of SLE patients in discovery cohort and validation cohort.

Characteristics	SLE in discovery cohort (n = 357)	SLE in validation cohort (n = 91)	*P*
Age (yr), mean/median	38/36	38/35	0.665 [a]
Gender, N (%)			
Female	334 (93.6%)	88 (96.7%)	0.252 [b]
Male	23 (6.4%)	3 (3.3%)	
Disease course (yr), mean/median	6/3	5/3	0.068 [c]
Organ involvement N (%)			
Renal	186 (52.1%)	42 (46.2%)	0.314 [c]
Hematological	106 (29.7%)	28 (30.8%)	0.843 [c]
Mucocutaneous	159 (44.5%)	44 (48.4%)	0.518 [c]
Musculoskeletal	94 (26.3%)	19 (20.9%)	0.286 [c]
Neuropsychiatric	16 (4.5%)	3 (3.3%)	0.587 [c]
Cardiorespiratory	71 (19.9%)	16 (17.6%)	0.612 [c]
Disease activity, median (IQR)			
SLEDAI	9 (9)	9 (10)	0.348 [c]
Laboratory tests, median (IQR)			
WBC ($*10^9$ L^{-1})	5.62 (3.76)	5.70 (4.96)	0.727 [c]
Hb (g L^{-1})	106.00 (29.00)	107.00 (28.00)	0.141 [c]
ESR	36.00 (46.00)	39.50 (45.50)	0.985 [c]
C3 (g L^{-1})	0.61 (0.40)	0.61 (0.43)	0.861 [c]
C4 (g L^{-1})	0.10 (0.10)	0.10 (0.09)	0.769 [c]
ANA positive/tested	315/318	77/77	0.448 [c]
dsDNA (IU mL^{-1})	28.01 (38.52)	22.69 (45.58)	0.898 [c]
24h urine protein (g per 24h)	0.98 (3.36)	0.95 (2.72)	0.436 [c]
Comorbility			
SS (n)	10	5	
APS (n)	9	2	
Corticosteroids, N(%)			
< 20 mg day^{-1}	100 (28.0%)	20 (21.9%)	
20-40 mg day^{-1}	104 (29.2%)	26 (28.6%)	
> 40 mg day^{-1}	79 (22.1%)	19 (20.9%)	
Unused	15 (4.2%)	7 (7.7%)	
Unknown	59 (16.5%)	19 (20.9%)	
Immunosuppressive agents [d], N (%)			
HCQ	155 (43.4%)	45 (49.5%)	
MMF	42 (11.8%)	10 (11.0%)	
CTX	27 (7.6%)	3 (3.3%)	
Others	94 (26.3)	17 (18.7%)	
Unused	101 (28.3%)	23 (25.3%)	
Unknown	28 (7.8%)	10 (11.0%)	

[a] is calculated by the Mann-Whitney U test.
[b] is calculated by χ^2 test.
[c] is calculated by two-tailed t-test.
[d] The immunosuppressive agents include hydroxychloroquine sulfate (HCQ), mycophenolate mofetil (MMF), cyclophosvnamide (CTX), and other agents like leflunomide (LEF), azathioprine (Aza), methotrexate (MTX), etc.

Table S3. Diagnostic performance of SLE by SMFs.

Machine learning algorithm [a]	Parameter [b]	Discovery (SLE/HC, 357/374)	Validation (SLE/HC, 91/93)
**Sparse learning**	AUC	0.950	0.992
	95% CI	0.935-0.965	0.983-1.000
	Sensitivity	0.860	0.890
	Specificity	0.920	1.000
**Decision tree**	AUC	0.486	0.533
	95% CI	0.444-0.528	0.450-0.617
	Sensitivity	0.451	0.516
	Specificity	0.521	0.418
**Logistic regression**	AUC	0.489	0.527
	95% CI	0.447-0.531	0.443-0.610
	Sensitivity	0.504	0.452
	Specificity	0.473	0.495
**Supporting vector machine (SVM)**	AUC	0.498	0.450
	95% CI	0.456-0.539	0.367-0.533
	Sensitivity	0.479	0.441
	Specificity	0.516	0.659
**K-nearst neighbors (kNN)**	AUC	0.544	0.523
	95% CI	0.503-0.586	0.440-0.607
	Sensitivity	0.602	0.613
	Specificity	0.487	0.341
**Random forest**	AUC	0.513	0.499
	95% CI	0.471-0.555	0.416-0.583
	Sensitivity	0.445	0.441
	Specificity	0.580	0.560

[a] The six different machine learning methods were conducted for SLE diagnosis.

[b] The parameter for evaluating the diagnostic performance includes area-under-the-curve (AUC) with 95% confidential interval (CI), sensitivity, and specificity.

Table S4. The diagnostic performance of sparse learning by tunning model number.

**Number** [a]	**Discovery cohort**		
	**AUC** [a]	**Sensitivity (%)**	**Specificity (%)**
**5**	0.95	86.8	92.3
**10**	0.95	88.2	92.0
**20**	0.95	86.8	92.5
**30**	0.95	87.4	92.3
**40**	0.95	86.3	93.1
**50**	0.95	86.9	92.7
**100**	0.95	86.0	92.0
**1000**	0.95	87.0	92.5

[a] The number referred to the diagnostic models obtained by the 5-fold cross-validation.

Table S5. Diagnostic performance of SLE by metabolic biomarker panel.

Organ involvement [a][b]	Parameter [b]	Neuropsychiatric	Hematological	Cardiorespiratory	Mucocutaneous	Musculoskeletal	Renal
Neuropsychiatric	AUC	-	-	-	-	-	-
	95% CI						
	Sensitivity						
	Specificity						
Hematological	AUC	0.54	-	-	-	-	-
	95% CI	0.36-0.72					
	Sensitivity	0.50					
	Specificity	0.73					
Cardiorespiratory	AUC	0.65	0.58	--	-	-	-
	95% CI	0.47-0.83	0.48-0.68				
	Sensitivity	0.56	0.48				
	Specificity	0.85	0.68				
Mucocutaneous	AUC	0.69	0.54	0.52	-	-	-
	95% CI	0.51-0.87	0.46-0.62	0.42-0.62			
	Sensitivity	0.74	0.62	0.11			
	Specificity	0.75	0.49	0.87			
Musculoskeletal	AUC	0.74	0.62	0.53	0.57	-	-
	95% CI	0.60-0.88	0.53-0.71	0.43-0.63	0.47-0.67		
	Sensitivity	0.74	0.74	0.64	0.35		
	Specificity	0.77	0.56	0.52	0.91		
Renal	AUC	0.61	0.60	0.53	0.60	0.67	-
	95% CI	0.43-0.79	0.50-0.70	0.43-0.63	0.52-0.68	0.59-0.75	
	Sensitivity	0.77	0.28	0.82	0.62	0.63	
	Specificity	0.55	0.91	0.34	0.62	0.73	

[a] refers to the six major organ involvements of SLE patients.

[b] The evaluation for diagnostic performance includes AUC with 95% CI, sensitivity, and specificity.

Table S6. Key *m/z* features from SMFs for identification of SLE patients from HCs.

Label	Metabolite	HMDB ID	*m/z*	Frequency[a]	Coefficient[b]	*p* [c]
**1#**	imidazoleacetic acid	HMDB0002024	164.90	99	0.14	0.0006
**2#**	2-hydroxyadipic acid	HMDB0000321	184.91	100	0.15	< 0.0001
**3#**	glucose	HMDB0000122	203.01	100	0.36	0.0002
**4#**	pseudouridine	HMDB0000767	267.01	100	-0.12	0.0131

[a] refers to the frequency of the *m/z* signal being selected in 100 sparse learning models.
[b] The scale factor, measuring the influence of specific *m/z* features in the model, was calculated as the coefficient.
[c] The *p*-value was obtained from the two-tailed t-test of specific *m/z* features between SLE patients and HCs.

Table S7. The matched *m/z* features within LDI-MS and LC-MS/MS.

**Label**	**1#**	**2#**	**3#**	**4#**
**Metabolite**	imidazoleacetic acid	2-hydroxyadipic acid	glucose	pseudouridine
**Molecular mass**	126.0429	162.0528	180.0634	244.0695
***m/z* in LDI-MS**	164.9	184.9	203.01	267.01
**Ions in LDI-MS**	$[M + K]^+$	$[M + Na]^+$	$[M + Na]^+$	$[M + Na]^+$
***m/z* in LC-MS**	125.0244	161.0482	179.0587	243.065
**Ions in LC-MS**	$[M - H]^-$	$[M - H]^-$	$[M - H]^-$	$[M - H]^-$
**Seperation mode**	HILIC	HILIC	HILIC	HILIC
**ESI mode**	negative	negative	negative	negative
**FC [a] in LDI-MS**	1.09	1.52	2.3	1.19
**FC [a] in LC-MS**	1.36	1.46	1.38	1.61

[a] Fold change (FC) was calculated by comparing the SLE patients with HCs.

Table S8. Diagnostic performance of SLE by metabolic biomarker panel.

Performance [a]	Discovery (SLE/HC, 357/374)	Validation (SLE/HC, 91/93)
AUC	0.877	0.800
95% CI	0.853-0.901	0.735-0.862
Sensitivity (%)	82.9	76.9
Specificity (%)	76.7	72.0

[a] The evaluation for diagnostic performance includes AUC with 95% CI, sensitivity, and specificity.

Table S9. The diagnostic performance of potential biomarkers.

**Label** [a]	**Performance**	**Discovery cohort**	**Validation cohort**
**1#**	AUC	0.71	0.65
	95% CI	0.68-0.74	0.62-0.68
	Sensitivity (%)	94.4	94.5
	Specificity (%)	37.3	32.3
**2#**	AUC	0.74	0.64
	95% CI	0.71-0.77	0.61-0.67
	Sensitivity (%)	73.2	59.3
	Specificity (%)	77.3	66.7
**3#**	AUC	0.81	0.83
	95% CI	0.78-0.84	0.80-0.86
	Sensitivity (%)	87.3	89.0
	Specificity (%)	49.3	73.1
**4#**	AUC	0.66	0.58
	95% CI	0.62-0.70	0.55-0.61
	Sensitivity (%)	53.5	83.5
	Specificity (%)	70.7	68.8

[a] Refers to the potential biomarker label in Table S6.

Table S10. The correlation coefficient between SLEDAI and average intensity.

Label [a]	Average SLEDAI	Average intensity	σ_{SLEDAI}	$\sigma_{Intensity}$	r [b]
**1#**	10.21849	1128.787	6.4853	1352.397	0.0061
**2#**	10.21849	444.8581	6.4853	446.8501	0.0756
**3#**	10.21849	2940.875	6.4853	3548.942	0.1214
**4#**	10.21849	138.2771	6.4853	108.8703	0.0394

[a] Refers to the potential biomarker label in Table S6.

[6] Using the equation: $\rho_{X,Y} = corr(X,Y) = \frac{cov(X,Y)}{\sigma_X \sigma_Y}$ and $r = \frac{1}{n-1}\sum\frac{(X-\mu_X)(Y-\mu_Y)}{\sigma_X \sigma_Y}$.

Table S11. The intensity of potential biomarkers.

Category	Label [a]	Intensity					
		1	2	3	4	5	Average [b]
HCs	1#	996.75	980.37	960.92	942.73	923.35	960.83 ± 29.17
	2#	182.66	178.09	175.48	173.34	174.23	176.76 ± 3.75
	3#	1548.79	1550.20	1581.60	1615.40	1658.42	1590.88 ± 3656.75
	4#	119.80	107.67	102.04	96.50	94.51	104.10 ± 10.17
SLE patients	1#	1059.51	1053.08	1046.51	1037.66	1016.60	1042.67 ± 16.67
	2#	287.20	277.50	265.20	258.80	250.80	267.90 ± 14.55
	3#	4169.95	3873.60	3618.98	3406.96	3214.24	3656.75 ± 377.51
	4#	138.23	128.28	120.98	117.45	114.50	123.89 ± 9.53

[a] refers to the potential biomarker label in Table S6.

[b] The averaged intensity with standard deviation were calculated according to five independent LDI MS experiments of 357 SLE patients and 374 HCs in the discovery cohort.

Table S12. Fold changes of the potential biomarkers of SLE.

Label [a]	FC [b]	FC [c]
**1#**	1.09	0.15
**2#**	1.52	0.31
**3#**	2.30	2.68
**4#**	1.19	0.34

[a] refers to the potential biomarker label in Table S6.

[b] Fold change (FC) was calculated by comparing the SLE patients with HCs at corresponding *m/z* features.

[c] FC was calculated by comparing the SLE patients with rheumatoid arthritis (RA) patients at corresponding *m/z* features.

Table S13. The intensity at potential biomarkers.

Category	Label [a]	Intensity					
		1	2	3	4	5	Average [b]
RA patients	1#	6279.00	7077.84	6741.91	6891.70	6648.21	6727.73 ± 298.89
	2#	994.89	944.09	861.71	789.41	690.99	856.22 ± 121.19
	3#	1337.85	1439.26	1309.88	1405.21	1321.62	1362.76 ± 56.49
	4#	348.54	389.39	369.29	371.22	340.51	363.79 ± 19.46
SLE patients	1#	1059.51	1053.08	1046.51	1037.66	1016.60	1042.67 ± 16.67
	2#	287.20	277.50	265.20	258.80	250.80	267.90 ± 14.55
	3#	4169.95	3873.60	3618.98	3406.96	3214.24	3656.75 ± 377.51
	4#	138.23	128.28	120.98	117.45	114.50	123.89 ± 9.53

[a] refers to the potential biomarker label in Table S6.

[b] The averaged intensity with standard deviation were calculated according to five independent LDI MS experiments of 27 RA patients and 357 SLE patients.

Table S14. Statistics of potential biomarkers.

**Label**	**Metabolite**	***m/z***	***p* [a]**
**1#**	imidazoleacetic acid	164.90	< 0.0001
**2#**	2-hydroxyadipic acid	184.91	0.0004
**3#**	glucose	203.01	0.0001
**4#**	pseudouridine	267.01	< 0.0001

[a] The *p* value was obtained from the two-tailed t-test of single *m/z* feature between 357 SLE patients and 27 RA patients.

Table S15. Demographic information and clinical features of RA patients and SLE patients selected by PSM.

Characteristics	SLE (n = 27)	RA (n = 27)	P_1 [a]	SLE (n = 10)	RA (n = 10)	P_2 [b]
Age (yr), mean (SD)	46.59 (11.85)	46.81 (9.21)	0.986 [c]	45.90 (7.29)	45.4 (8.77)	0.909 [c]
Gender, N (%)						
Female	25 (92.6)	25 (92.6)	1 [d]	9	9	1 [d]
Male	2 (7.4)	2 (7.4)		1	1	
Steroid dose, mean (SD)	2.22 (4.62)	2.22 (3.42)	1 [c]		——	
Steroid (%)						
Not used	21 (77.8)	17 (63.0)	0.371 [e]	10	10	1 [e]
used	6 (22.2)	10 (37.0)		0	0	
MTX (%)						
Not used	22 (81.5)	13 (48.1)	0.023 [e]	10	10	1 [e]
used	5 (18.5)	14 (51.9)		0	0	
LEF (%)						
Not used	26 (96.3)	19 (70.4)	0.028 [e]	10	10	1 [e]
used	1 (3.7)	8 (29.6)		0	0	
HCQ (%)						
Not used	22 (81.5)	22 (81.5)	1 [e]	10	10	1 [e]
used	5 (18.5)	5 (18.5)		0	0	

[a] is calculated based on the 54 patients (SLE/RA, 27/27) who are selected by PSM method.

[b] is calculated based on the 20 patients (SLE/RA, 10/10) who treatment naïve.

[c] is calculated by the Mann-Whitney U test.

[d] is calculated by χ^2 test.

[e] is calculated by t-test.

[f] The immunosuppressive agents include hydroxychloroquine sulfate (HCQ), methotrexate (MTX), and leflunomide (LEF).

Table S16. Clinical features of HCs and SLE patients for illustrating medication influence.

Characteristics	SLE with nomedications (n = 48)	SLE with medications (n = 48)	HCs (n = 48)	*P1* [a]	*P2* [b]
Age (yr), mean/median	38/34	38/34	38/34	0.971 [c]	0.783 [c]
Gender, N (%)					
Female	44 (91.7%)	44 (91.7%)	44 (91.7%)	1.000 [d]	1.000 [d]
Male	4 (8.3%)	4 (8.3%)	4 (8.3%)		

[a] refers to the statistical results between SLE patients who received no medications and HCs.

[b] refers to the statistical results between SLE patients who received no medications and SLE patient with medications.

[c] is calculated by the Mann-Whitney U test.

[d] is calculated by χ^2 test.

Table S17. The comparison between the prior serum metabolomic studies and the present study in SLE.

No.	Sample size (SLE/HC)	Validation cohort[a]	Detection platform[b]	Statistical methods[c]	Metabolites[d]	Diagnostic performance[e]			Ref
						AUC	Sen (%)	Spe (%)	
This work	448/467	Yes	LDI MS	Sparse learning	Imidazoleacetic acid, 2-hydroxyadipic acid, glucose and pseudouridine	0.950	86.0	92.0	—
1	64/35	No	^{1}H-NMR	PCA, PLS-DA	N-acetyl glycoprotein (NAG)<br>Valine, tyrosine, phenylalanine, lysine, isoleucine, histidine, glutamine, alanine, citrate, creatinine, creatine, pyruvate, high-density lipoprotein, cholesterol, glycerol, formate,	—	60.9	97.1	Ref [11]
2	58/9	Yes	GC-MS,LC-MS	Random Forest	MDA, gamma-glutamyl peptides, GGT, leukotriene B4 and 5-HETE	> 0.87	—	—	Ref [12]
3	30/18	No	GC-MS	PCA	Glucose, urea, cystine, threonine, naproxen, lysine, fumaric acid, malic acid, methionine, tyrosine, theobromine, alanine, asparagine, threonic acid, hidtinde, caffeine, lactic acid, cysteine, citric acid, tryotophan	0.606-0.759	—	—	Ref [13]
4	22/30	No	NMR	PLS-DA	Glucose, acetate, NAG,<br>leucine, valine, alanine, glutamic acid, citrate, choline, proline, glycine, lactate,<br>Lipids (L1-L9)	> 0.76	—	—	Ref [14]
5	80/57	No	GC-MS	PCA, PLS-DA	1-Monopalmitin, Cystine, 2-Hydroxyisobutyrate, 1-Monolinolein, Glutamate, 1-Monoolein, Methionine, 4-Hydroxybutyrate, Mannose, Arachidonic acid, Fumarate, Lysine, Histidine, Serine, Myo-inosito-1-phosphate, Alpha-aminobutyrate, Alpha-tocopherol, Glucose, Leucine, Tyrosine, Fructose, Glycine, Valine, 2-Hydroxyisovalerate, Alanine, Proline, Asparagine, Glycerol, Pyroglutamarate, Glutamine, Aminomalonate, Isoleucine, Gluconic acid-lactone, Tryptophan, 2-Keto-3-methylvalerate, Threonine, Threonate, Beta-D-Methylglucopyranoside, 2-Ketoisocaproate, Oleic acid, Aspartate	0.764-0.924	—	—	Ref [15]
6	32/28	No	LC-MS	OPLS-DA	Sorbitol, theophylline, oxidized glutathione, capric acid, 3-Indolepropionic acid, norvaline, hippuric acid, sphingosine, cortisol, NAG, glucose 6-phosphate, riboflavin, taurine, creatinine	—	87.5	67.9	Ref [16]
7	16/30	No	NMR	PCA, PLS-DA	Lipoproteins and lipids, acetate, amino acids	0.76-1.00	—	—	Ref [17]
8	17/17	No	LC-MS	PCA	2-counmaric acid, acetylcholine, guanidonoproplonic acid, Galacturonic acid, inosine, rac-glycerol 3-phosphoate, S-3-Amino-4-phenylbutyric acid, S-3-Amino-5-methyylhexanoic acid, trimethylamine N-oxide, Xanthine,	0.75-0.875	—	—	Ref [18]

					arginine, asparagine, glutamic acid, histidine, serine, homoproline, homothreonine, homovaline, allopurinol, dimefline, sorbitol, dulcitol, flonicamid, leupeptin, maltitol, mycophenolic acid, prednisolone, ADP, caffeine, hydrocortisone, itaconic acid, serotonin				
9	68/55	Yes	LC-MS	PLS-DA	MG 20:2 and L-pyroglutamic acid, arachidonic acid, sphingomyelin (SM) 24:1, monoacylglycerol (MG) 17:0, lysophosphatidyl ethanolamine (lysoPE) 18:0, lysoPE 16:0, lysophosphatidyl choline (lysoPC) 20:0, lysoPC 18:0 and adenosine	0.955	97.2	83.3	Ref [19]

[a] refers to the situation if the corresponding study has an independent validation cohort.

[b] refers to the detection platform utilized in the corresponding study, including laser desorption/ionization mass spectrometry (LDI MS), nuclear magnetic resonance (NMR), gas chromatography mass spectrometry (GC-MS), and liquid chromatography mass spectrometry (LC-MS).

[c] refers to the statistical methods utilized in the corresponding study, including sparse learning, principal component analysis (PCA), partial least squares discriminant analysis (PLS-DA), and orthogonal partial least squares discriminant analysis (OPLS-DA).

[d] refers to the metabolite biomarker identified between SLE patients and HCs. The red color and blue color indicate the up-regulated metabolites and down-regulated metabolites, respectively.

[e] The diagnostic performance includes area-under-the-curve (AUC), sensitivity (Sen), and specificity (Spe).
